# A Multi-Omics Analysis Suggests Links Between the Differentiated Surface Metabolome and Epiphytic Microbiota Along the Thallus of a Mediterranean Seaweed Holobiont

**DOI:** 10.3389/fmicb.2020.00494

**Published:** 2020-03-25

**Authors:** Benoît Paix, Nathan Carriot, Raphaëlle Barry-Martinet, Stéphane Greff, Benjamin Misson, Jean-François Briand, Gérald Culioli

**Affiliations:** ^1^EA 4323, Matériaux Polymères Interfaces Environnement Marin, Université de Toulon, Toulon, France; ^2^UMR 7263, Aix Marseille Université, CNRS, IRD, Avignon Université, Institut Méditerranéen de Biodiversité et d’Ecologie Marine et Continentale, Station Marine d’Endoume, Marseille, France; ^3^UMR 7294, Université de Toulon, Aix Marseille Université, CNRS, IRD, Mediterranean Institute of Oceanography, Marseille, France

**Keywords:** seaweed surface, holobiont, microbial community, metabolomics, multi-omics analysis

## Abstract

Marine macroalgae constitute an important living resource in marine ecosystems and complex ecological interactions occur at their surfaces with microbial communities. In this context, the present study aimed to investigate how the surface metabolome of the algal holobiont *Taonia atomaria* could drive epiphytic microbiota variations at the thallus scale. First, a clear discrimination was observed between algal surface, planktonic and rocky prokaryotic communities. These data strengthened the hypothesis of an active role of the algal host in the selection of epiphytic communities. Moreover, significant higher epibacterial density and α-diversity were found at the basal algal parts compared to the apical ones, suggesting a maturation gradient of the community along the thallus. In parallel, a multiplatform mass spectrometry-based metabolomics study, using molecular networking to annotate relevant metabolites, highlighted a clear chemical differentiation at the algal surface along the thallus with similar clustering as for microbial communities. In that respect, higher amounts of sesquiterpenes, phosphatidylcholines (PCs), and diacylglycerylhydroxymethyl-*N*,*N*,*N*-trimethyl-β-alanines (DGTAs) were observed at the apical regions while dimethylsulfoniopropionate (DMSP) and carotenoids were predominantly found at the basal parts of the thalli. A weighted UniFrac distance-based redundancy analysis linking the metabolomics and metabarcoding datasets indicated that these surface compounds, presumably of algal origin, may drive the zonal variability of the epibacterial communities. As only few studies were focused on microbiota and metabolome variation along a single algal thallus, these results improved our understanding about seaweed holobionts. Through this multi-omics approach at the thallus scale, we suggested a plausible scenario where the chemical production at the surface of *T. atomaria*, mainly induced by the algal physiology, could explain the specificity and the variations of the surface microbiota along the thallus.

## Introduction

Marine macroalgae are major contributors of marine coastal biodiversity and considered as engineers in such ecosystems. They form natural habitats acting as ecological niches for surrounding organisms, in particular for many epiphytic life-forms found at their surfaces (Wahl et al., 2012). With surface densities recorded from 10^2^ to 10^7^ cells.cm^–2^ across different seaweeds, epiphytic bacteria are the main contributors to the microbial communities associated with algal surfaces (Bengtsson and Øvreås, 2010; Hollants et al., 2013).

Several studies have already highlighted the importance of inter-kingdom interactions, which can be essential for the physiology of all partners (Wichard et al., 2015). In that respect, some bacterial strains have been shown to be involved in the morphogenesis of the green alga *Ulva mutabilis*. This seaweed releases dimethylsulfoniopropionate (DMSP) into the surrounding waters which acts as a chemoattractant food source signal for the *Roseovarius* sp. MS2 strain. Once the signal is detected, the bacterial cells use the glycerol boundary layer as carbon source and promote the morphogenesis of two *Ulva* spp. (Wichard and Beemelmanns, 2018; Kessler et al., 2018). Conversely, several other studies have demonstrated the detrimental effect of some epiphytic bacteria which can negatively affect the host fitness. In the case of the red seaweed *Delisea pulchra*, the occurrence of pathogenic bacteria causing thallus bleaching was linked with a decrease of the algal chemical defenses (halogenated furanones acting as quorum sensing inhibitors) observed during the summer period (Wright et al., 2000; Campbell et al., 2011; Case et al., 2011). Epiphytes associated to macroalgae can even show evolutionary adaptation to macroalgal niches through, for example, their ability to degrade algal cell walls (Gobet et al., 2018). The close interactions between seaweeds and their surface-associated microbiota lead to consider these biological systems as holobionts (Egan et al., 2013).

With the increasing number of studies and models showing such interactions, it has been shown that the chemical production at the surface of algae may represent one of the main parameter driving the dynamic of epiphytic microbial communities (Nylund et al., 2010; Lachnit et al., 2013; Saha et al., 2014). In addition, environmental parameters also seem to be involved in the shaping of surface microbiota of seaweed holobionts such as *Fucus vesiculosus, Ecklonia radiata*, *Caulerpa prolifera*, *Caulerpa cylindracea*, *Macrocystis pyrifera*, *Delisea pulchra*, and *Sargassum muticum* (Stratil et al., 2013, 2014; Marzinelli et al., 2015, 2018; Zozaya-Valdés et al., 2016; Aires et al., 2018; Minich et al., 2018; Morrissey et al., 2019; Qiu et al., 2019). Nevertheless, while the microbial communities associated with the surface of algae are increasingly studied, the chemical composition of the surface of the algal hosts and the variations of the metabolic production at the thallus scale have been only rarely investigated to date.

In this context, the aim of this study was to evaluate to what extent metabolites produced by an algal holobiont at its own surface could lead to changes in the epiphytic microbiota community at the thallus scale. To our knowledge, this study was the first to couple intra-thallus variations of the surface metabolome of an algal host, thus taking into account physiological differentiations such as algal growth, and those of its epiphytic communities. The seaweed holobiont model selected for this study, *Taonia atomaria* (Woodward) J. Agardh, is an annual photophilic marine Phaeophyceae widely reported along the Mediterranean and Northwestern Atlantic coasts. This intertidal seaweed is commonly found from February to July on infralittoral rocky habitats of the French Mediterranean coasts (Sala and Boudouresque, 1997). Several compounds isolated from extracts of *T. atomaria*, and more specifically expressed at its surface, have been previously shown to display anti-adhesion properties and thus could be involved in the selection of specific epibacterial communities (Othmani et al., 2016a, b). More recently, a clear temporal co-variation of the structure of the epibacterial communities and surface metabolites has been highlighted for *T. atomaria* collected on the French Mediterranean coasts (Paix et al., 2019).

In the present study, metabarcoding and flow cytometry were performed to determine whether prokaryotic communities found at the surface of *T. atomaria* were specific and whether they varied at the thallus scale. Thus, the specificity of surface prokaryotic communities was evaluated using, in addition to algal samples, surrounding water and biofilms formed on nearby small rocks. Intra-thallus variability was assessed dividing each thallus into three parts from the base to the apex. Then, at the same thallus scale, we studied in parallel the variations of the endometabolome and surface metabolome of the alga using a multi-platform mass spectrometry-based metabolomics approach [LC-ESI-(+)-MS, LC-ESI-(−)-MS and GC-MS], with the aim of increasing the annotation of relevant metabolites through the use of molecular networking. The use of a specific analytical methodology previously validated on this algal model allowing the extraction and the annotation of a wide range of surface molecules (Othmani et al., 2016a; Paix et al., 2019), as well as the ability to compare the endometabolome and surface metabolome of *T. atomaria*, was of crucial interest to gain insight the biological origin of the compounds implied in the intra-thallus differentiation of the surface extracts. In light of these results, a UniFrac distance-based redundancy analysis coupling the resulting multi-omics datasets was conducted to understand to what extent the intra-thallus variations of algal surface metabolites could shape the epiphytic microbiota of *T. atomaria*. To test whether the potential variations along the thalli were not restricted to a specific geographical area, this study was conducted on two sampling sites on the South-Eastern French Mediterranean coasts.

## Materials and Methods

### Sampling Strategy

The sampling was performed on June 2017 by scuba diving at two sites located along the French Mediterranean coasts: Tamaris (La Seyne-sur-Mer; 43°5′35.56′′N, 5°54′31.81′′E) and Carqueiranne (43°5′12.41′′N, 6°5′3.26′′E). Thalli of *T. atomaria* (Woodward) J. Agardh (Class: Phaeophyceae, order: Dictyotales, family: Dictyotaceae) were collected by carefully detaching holdfasts from rocky substrates (1 m depth). Algal samples were stored in sterile bags filled with surrounding seawater. For both sampling sites, different individuals harvested on the same rocky substrate were considered as biological replicates. In addition to seaweed samples, triplicates of rocks (type of substrates chosen haphazardly) and surrounding seawater (5 L) were also collected. From the point of collection, samples were transported in a cool box maintaining the seawater temperature and treated at lab within 1 h as previously described (Paix et al., 2019).

The global analytical workflow used in this study was described in Supplementary Figure S1. More precisely, once at lab, only thalli measuring 9 ± 0.5 cm length were considered. To ensure multi-omics cross-comparison, metabarcoding and metabolomics analyses were performed for each replicate on different fronds from the same thallus. Distinct thalli were used for cytometry and confocal microscopy. For each thallus, fronds were divided into three parts of equal length (3 ± 0.2 cm), defined as basal, median and apical parts (Supplementary Figures S2A,B). These parts were separated with a sterile scalpel and photographed to estimate their surface with the Mesurim pro software (v. 3.4). Before flow cytometry and molecular approaches, microbiota of *T. atomaria* were sampled by scraping the surface of the three parts of each replicate of thalli (*n* = 3) with a sterile scalpel as previously described (Paix et al., 2019). Biofilms on rocks were sampled using the same methodology and seawater samples (5 L) were filtered on 0.2 μm filters (Millipore-Merck, Darmstadt, Germany).

### Confocal Laser Scanning Microscopy (CLSM)

Triplicates of thalli of *T. atomaria* were fixed during 30 min in a 3.7% formaldehyde solution, then washed three times with artificial seawater (ASW). Squares of basal, median, and apical parts were dissected in each pre-fixed thallus and stained individually in a 24-well plate. Staining was performed in the dark, with DAPI (4 μg.mL^–1^; Sigma-Aldrich-Merck, Darmstadt, Germany) for 20 min at room temperature. After three washes in ASW, squares were mounted on microscope slices with one drop of ProLong Diamond Antifade (ThermoFisher Scientific, Waltham, MA, United States) and incubated for 24 h at room temperature in the dark. Confocal images were acquired with a 20×/0.75NA objective on a Zeiss Confocal LSM 510 Meta. Individual tracks were set as follow: laser 405 nm – BP 405-480 IR; laser 488 nm – LP 650, to respectively acquire DAPI and chlorophyll signals.

### Quantitative Flow Cytometry Analyses

Flow cytometry analyses were used to assess the epiphytic heterotroph densities at the surface of the algal samples. Analyses were conducted only for samples of *T. atomaria* collected in Tamaris, since not enough algal replicates were available at Carqueiranne. Replicates of samples (*n* = 3) were fixed in 4 mL of 1% glutaraldehyde-sterile filtered seawater solution and directly conserved at −80°C until analysis. Aggregated cells were dissociated according to Pollet et al. (2018) with an optimized sonication time of 2 min. Heterotrophic prokaryotes were stained using SYBR green I (Invitrogen, Carlsbad, CA, United States) and enumerated using a BD Accuri C6 flow cytometer (BD Biosciences, San Jose, CA, United States) as previously described (Pollet et al., 2018) (Supplementary Figure S3). Results were expressed as densities of cells per cm^2^ using the measured surface of each thallus part.

### DNA Extraction, 16S rRNA Gene Amplification and Sequencing

DNA extraction of biofilm samples was performed using the DNeasy PowerBiofilm kit (MoBio, Qiagen, Germantown, MD, United States) and samples were conserved at −80°C. DNA extraction of filtered seawater samples was performed using the SA-Gen method described in Vasselon et al. (2017). After DNA extraction, the V4-V5 region of 16S rRNA gene was amplified using 515F-Y and 926R primers (Parada et al., 2016), following the PCR protocol from Pollet et al. (2018). Amplicons were sent to GeT Platform (Toulouse, France) for MiSeq Illumina sequencing (2 × 250 bp).

### 16S rRNA Gene Metabarcoding Data Processing and Analysis

16S rRNA gene reads were processed using the FROGS workflow under Galaxy environment (Escudié et al., 2018). Sequences were quality filtered by removing those for which primers sequences were not present. The primer search accepts 10% of differences. Primers sequences were then removed in the remaining sequence using “cutadapt.” Then, merged sequences with length below 300 pb and above 500 pb were removed. Clustering step was performed using SWARM with a clustering aggregation distance set to 3 (Mahé et al., 2014). Chimeric sequences were removed *de novo* using VSEARCH (Rognes et al., 2016). Rare OTUs representing less than 0.005% of all sequences were removed. OTUs were affiliated with the silva132 16S rRNA gene database. The final matrix was obtained by removing all sequences affiliated to 16S rRNA gene from chloroplasts and mitochondria, and by performing a rarefaction to the minimum library size using the “phyloseq” R package (McMurdie and Holmes, 2013). Mean percentages of sequences affiliated to chloroplasts and mitochondria were 36.3 and 1.2%, respectively. α-Diversity was estimated using the number of OTUs, Chao1 and Shannon indexes. β-Diversity was analyzed with a non-metric multidimensional scaling (NMDS), using weighted UniFrac distances, allowing to consider phylogenetic distances between OTUs. These analyses were performed using the “phyloseq” R package and graphical outputs were generated using the “ggplot2” R package. Discriminant analyses were performed to determine specific taxa of each group of samples for both sampling sites, using the LEfSe algorithm in the Galaxy environment with a LDA threshold set to 4. SIMPER analyses were performed with the “vegan” R package to identify the relative contribution of the most relevant prokaryotic genera involved in the dissimilarities between: (i) seawater and algal samples, (ii) rocky biofilms and algal samples, and (iii) basal and apical zones of the thalli. Co-occurrence networks (OTUs–OTUs) were built using the Cytoscape App CoNet (Faust and Raes, 2016). Data were processed using Pearson and Spearman correlation, with Bray–Curtis and Kullback–Leiber dissimilarity distances, and using a threshold of 200 edges. Venn diagrams were used to identify the relative percentage of sequences shared between the different groups of samples and were generated using the Venn webtool^1^. An OTU was considered as common to different groups of samples when it was found at least in one triplicate of each group of samples.

### Extraction and Samples Preparation for Metabolomics Approaches

Extraction of surface metabolomes (surface extracts) was performed by dipping each algal part in 5 mL of liquid chromatography-mass spectrometry (LC-MS) grade methanol (MeOH; VWR, Fontenay-sous-Bois, France) during 5 s, according to Othmani et al. (2016a) and Paix et al. (2019). This protocol has been previously developed in order to preserve the integrity of the outer membrane cell of *T. atomaria*. Three algal replicates were used for Carqueiranne (*n* = 3) and five for Tamaris (*n* = 5). For this extraction step, particular care was taken to prevent any contact between the solvent and cut ends of thalli to prevent any release of intracellular metabolites (Supplementary Figure S2A). The algal endometabolomes (total extracts) were then obtained by dipping the thallus parts previously used for the extraction of surface metabolomes in 5 mL of MeOH during 24 h in the dark (see Supplementary Figure S2A). Six experimental blanks for each type of extracts were also prepared during this procedure. The resulting surface and total extracts were dried under N_2_ flow and stored in 8 mL vials at −20°C under inert atmosphere (Argon) until analysis.

Samples were prepared by solubilizing the resulting extracts in 1 mL of MeOH for LC-MS analyses and 1 mL of dichloromethane (CH_2_Cl_2_) for GC-MS analyses. Ten quality control samples (QCs) were prepared by mixing all the samples at equimolar concentrations. Additionally, two experimental blanks (only MeOH or CH_2_Cl_2_) were also prepared. Samples and experimental blanks were randomly injected and a QC was injected every five samples. Analytical blanks were injected at the beginning and at the end of the injection sequence.

The LC-ESI-MS analyses were performed on a UHPLC-HRMS system (Dionex Ultimate 3000 Rapid Separation; Thermo Fisher Scientific) coupled with a QToF Impact II mass spectrometer (Bruker Daltonics, Bremen, Germany) in positive and negative ionization modes. The separations were carried out with an analytical core-shell reversed-phase column (150 × 2.1 mm, 1.7 μm, Kinetex Phenyl-Hexyl; Phenomenex, Le Pecq, France). More details are given in Supplementary Information.

The GC-MS analyses were performed on a 7890B GC system equipped with a 7693 autosampler and coupled to a 5977A MSD mass selective detector (Agilent Technologies, Palo Alto, CA, United States) using to the same methodology described in Gaubert et al. (2019), as detailed in Supplementary Information.

### Metabolomics Data Processing and Analysis

LC-MS raw data obtained in positive and in negative modes were respectively converted into netCDF files using DataAnalysis (v. 4.3; Bruker, Germany) and processed with XCMS using Workflow4Metabolomics (W4M) under the Galaxy environment^2^ (Giacomoni et al., 2015). GC-MS raw data were converted into netCDF files using MSD ChemStation (v. F.01.00.1903) and were processed with the R package “eRah” (Domingo-Almenara et al., 2016). Parameters used for peak picking, alignment of peaks and gap filling are listed in Supplementary Table S1. Following each workflow, the three data matrices were submitted to three filtering steps using an in-house script on R. Each step of the script consisted in removing successively experimental and analytical bias according to signal/noise ratio (using blanks), coefficient of variation (using QCs), and coefficient of correlation (using samples). After filtration of the three data matrices, a chemodiversity estimation was calculated using the Shannon index. For multivariate analyses, the three resulting data matrices were analyzed using the Metaboanalyst 3.5 online webtool^3^ (Chong et al., 2018). These data were log_10_-transformed, mean-centered and normalized using the sum of the chromatographic peak areas. This method of normalization was found to be identical to that based on the algal surface areas, since a proportional relationship was observed between the sum of the chromatographic peak areas and the algal surfaces (linear regression: *R*^2^ > 0.9, see Supplementary Table S2). The same results, in terms of significance for ANOVA, *post hoc* tests and VIP ranks, were observed using both normalization methods. Data were then analyzed using principal component analysis (PCA), followed by partial least-square discriminant analysis (PLS-DA) allowing to reveal features discriminating samples between each part of the thalli for both sites. These VIP (Variable Importance in Projection) features were selected according to their VIP score (>2) and their significance, and particular attention was paid to their annotation.

### Annotation Strategy for Metabolomics Analysis

For LC-ESI-MS data, the methodology of annotation described in Paix et al. (2019) was applied and is detailed in Supplementary Information. In brief, isolated compounds and commercial standards were used, together with public databases, for the annotation of selected MS/MS spectra. Moreover, molecular networks were used through the GNPS platform^4^ to facilitate the dereplication procedure by comparing MS/MS spectra obtained in both positive and negative modes. For GC-MS, purified standards, Wiley 2008 and NIST 2011 databases together with the calculation of the van Den Dool and Kratz retention indices were used to annotate the dataset (van Den Dool and Kratz, 1963).

### Integration of Surface Metabolome and Surface Microbiota Datasets

Metabolites were used as explaining factors to investigate whether they could explain the variations of the structure of microbiota communities along thalli. A distance-based redundancy analysis (db-RDA) was conducted with the weighted UniFrac distance matrix from the 16S rRNA gene dataset and normalized concentrations of significant, discriminant and annotated surface compounds. This analysis was performed using the “phyloseq” and “vegan” R packages with the *capscale()* function using the methodology described by Shankar et al. (2017). Since for each replicate the same thallus was used for both metabarcoding and metabolomics analyses, the correspondence between each replicate and between both analyses was considered in the correlation between both datasets.

### Statistical Tests and Cross Validations

One-way ANOVA followed by HSD Tukey’s tests were used to evaluate the significance of density, diversity and specific metabolites across the different groups of samples (geographical sites and thallus parts). One-way ANOVA followed by HSD Tukey’s tests were performed using respectively the *aov()* and *HSD.test()* functions from the “ade4” and “agricolae” R packages (Dray and Dufour, 2007; de Mendiburu, 2019). Following NMDS and PCA, discriminations between the different groups of samples were statistically tested with a PERMANOVA and a multivariate pairwise test using the *adonis()* and *pairwise.perm.manova()* functions with the “vegan” and “RVAideMemoire” packages, respectively. Multivariate pairwise tests were conducted with weighted UniFrac and Euclidean distances for metabolomics and metabarcoding datasets, respectively. PLS-DA were subjected to cross-validations using the MetaboAnalyst tool. db-RDA model for datasets integration was statistically tested using the *anova.cca()* function.

## Results

### Confocal Laser Scanning Microscopy

Confocal laser scanning microscopy images only allowed to have a global visualization of the seaweed surface coverage without any ambition to identify or quantify organisms. These images were analyzed regarding individuals and merged channels (red: chlorophyll auto-fluorescence, gray: DAPI staining) (Supplementary Figure S4). Chlorophyll emission showed several diatom-like structures. With DAPI staining, a relatively high density of filamentous structures, assigned to filamentous bacterial cells according to their size (50–100 μm of length and 2 μm of diameter), was observed. Those filaments did not reveal any overlapping signal with chlorophyll auto-fluorescence, supporting the fact that they could be whether heterotrophic filamentous bacteria or fungi hyphae. Interestingly, these structures were mainly attached at the interface between algal cells. Finally, when comparing the different thallus parts, no clear pattern of surface colonization could be determined, as both diatom-like and filamentous bacteria-like structures were mainly observed indiscriminately on all algal samples.

### Quantification of Epiphytic Cells Densities by Flow Cytometry

**FIGURE 1:**
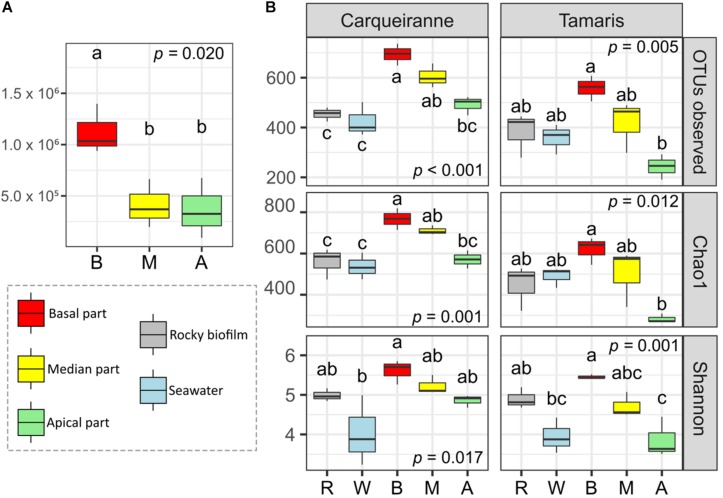
Variations of cells densities along the surface of *Taonia atomaria* and differences in α-diversity between algal parts, rocky biofilms and seawater collected in two Mediterranean sites (Carqueiranne and Tamaris). **(A)** Heterotrophic prokaryotes cell densities (cells.cm^–2^) variations of the different parts [basal (B), median (M), and apical (A)] of the thallus surface of *T. atomaria* in Tamaris. **(B)** Variations of several α-diversity indexes of prokaryotic communities at the surface of thallus parts [basal (B), median (M), and apical (A)] of *T. atomaria*, in rocky biofilms (R) and in surrounding water (W). Boxplots correspond to whisker plots (*n* = 3) showing the lowest, median and highest values. *P*-values and “a,” “b,” “c” indexes correspond to the results of one-way ANOVA analyses and *post hoc* tests (HSD Tukey’s test), respectively.

Flow cytometry analyses were only performed on thalli collected in Tamaris and showed significant higher densities of heterotrophic prokaryotes at the basal algal parts (Figure 1A, *p* = 0.02). Density of epiphytic heterotrophic prokaryotes increased by an average of 189% from the apical/median parts (0.39 × 10^6^ cells.cm^–2^) to the basal ones (1.12 × 10^6^ cells.cm^–2^). No significant difference was observed between the median and the apical parts (*post hoc* test).

### Diversity and Structure of Bacterial Communities

Rarefaction curves reached a plateau indicating a good coverage of the global diversity for all samples (Supplementary Figure S5). Normalization was performed at the smallest sequenced samples, i.e., 6939 sequences.

α-Diversity was measured using the number of OTUs, Chao1, and Shannon indexes (Figure 1B). When considering the different parts of *T. atomaria* collected in Tamaris, all indexes showed significant higher values for the basal parts in comparison with the apical ones (*p* < 0.05). No significant difference could be observed between the median parts and the two other algal parts. For samples collected in Carqueiranne, the same significant trend was only observed for the richness indexes (number of OTUs and Chao1). Water samples showed a lower α-diversity than that of basal parts for the Shannon index at both sites and the richness indexes only at Carqueiranne (*p* < 0.05). Biofilm samples collected on rocky substrates showed significant lower richness indexes compared to those of the basal and median parts of algal thalli collected in Carqueiranne (*p* < 0.05).

**FIGURE 2:**
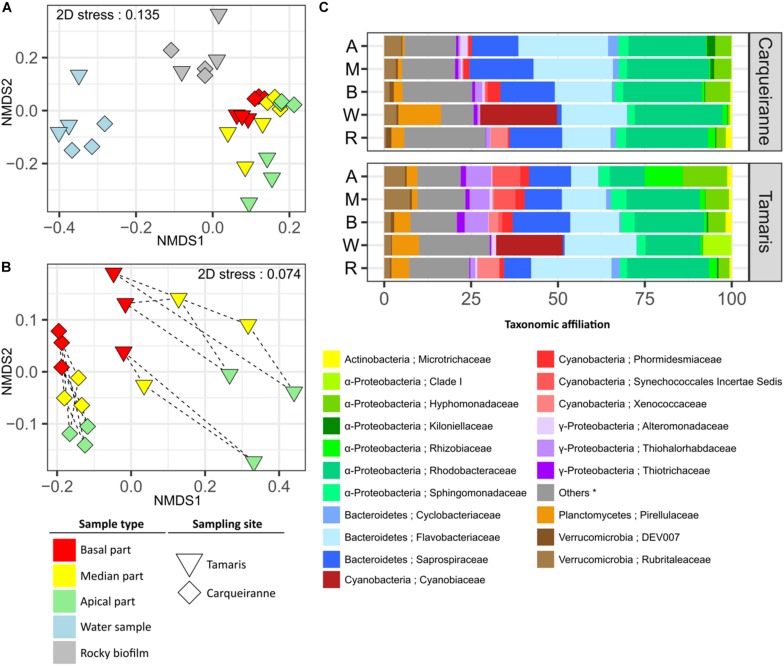
Structure and β-diversity of epibacterial communities at the surface of *T. atomaria*, in rocky biofilms and in seawater collected in two Mediterranean sites (Carqueiranne and Tamaris). NMDS plots based on weighted UniFrac distance for all samples **(A)** and algal samples only **(B)**. Dashed lines grouped the different parts of a same individual thallus. **(C)** Relative percentages of each bacterial family across each group of samples (*n* = 3). Labels A, M, B, W, and R corresponded to apical, median and basal algal parts, water samples and rocky biofilms, respectively. (^∗^) “Others” correspond to unaffiliated families and/or families with a relative percentage below 1%.

β-Diversity was analyzed using weighted UniFrac distance. The resulting NMDS plot (Figure 2A) showed a distinct clustering pattern between prokaryotic communities from surrounding seawater, biofilms on rocky substrates and biofilms on *T. atomaria* surface (Supplementary Tables S3, S4). Despite the rationale was not to compare both sites, the corresponding samples appeared as two separate groups (Supplementary Tables S3, S4). When focusing on algal samples (Figure 2B), the basal and apical zones were significantly discriminated but both did not appear significantly different from the median parts (Supplementary Tables S3, S4). Thalli from Tamaris showed a higher dissimilarity between parts comparing to those collected in Carqueiranne. A lower dissimilarity was observed between the basal parts and the other algal parts at both sites. For both sites, weighted UniFrac distances between biofilms formed on rocky substrates and basal parts were significantly lower than those between biofilms on rocky substrates and apical parts (Supplementary Figure S6).

Bacterial community structure in water samples was clearly distinct from that of other samples with several discriminant orders, such as Synechococcales, Myxococcales, and Cellvibrionales for Tamaris, and Pirellulales for Carqueiranne (Supplementary Figures S7, S8). More particularly for the Synechococcales, the dominance of Cyanobiaceae clearly appeared at both sites (Figure 2C). The main representing genus, *Synechococcus*, contributed to 11 and 9% of the overall dissimilarities with algal samples from Carqueiranne and Tamaris, respectively (Supplementary Tables S5, S6). At Carqueiranne, *Polaribacter* (Flavobacteriaceae) showed significant higher percentages in water samples and contributed to 4% of the dissimilarity between water and algal samples at this site (Supplementary Table S5). At Tamaris, the SAR11 Clade Ia was significantly present in water samples and contributed to 4% of the dissimilarity with algal samples (Supplementary Table S6). With a dominance of Saprospiraceae (8 to 19% of sequences), Rhodobacteraceae (10 to 24% of sequences), and Flavobacteriaceae (from 8 to 26% of sequences), Proteobacteria (mainly α-Proteobacteria) and Bacteroidetes were the two main phyla observed in epiphytic algal and rocky communities (Figure 2C). In particular, an unidentified genus from the Saprospiraceae was predominantly found on algal samples and contributed significantly (3%) to the dissimilarity with water samples at both sites (Supplementary Tables S5, S6).

The genera *Algitalea* (Flavobacteriaceae) at Carqueiranne and *Granulosicoccus* (Thiohalorhabdaceae) at Tamaris were observed in significant higher abundance on algal samples compared with rocky samples. These genera contributed to 3 and 5% of the dissimilarity between both sample groups, respectively (Supplementary Tables S7, S8). The Nostocales, and more precisely the Xenococcaceae family with the genus *Pleurocapsa*, were found as biomarkers of rocky biofilms at Tamaris (Supplementary Figure S7). *Pleurocapsa* notably contributed to 2% of the dissimilarity between rocky and algal samples from Tamaris (Supplementary Table S8).

Differences between algal parts were mainly due to genera among Rhodobacteraceae (*Loktanella*), Rubritaleaceae (*Rubritalea*) and especially Flavobacteriaceae (*Algitalea* and *Croceitalea*) which were found with higher percentages in the apical parts at Carqueiranne (Supplementary Table S9 and Figure 2C). The families Kiloniellaceae, Alteromonadaceae, and Rubritaleaceae were identified as biomarkers of the apical parts (Supplementary Figure S8). More precisely, the genus *Rubritalea* represented 2% of the dissimilarity with the basal parts as mentioned above. Differences among the algal parts were mainly based on taxa belonging to the α-Proteobacteria for Tamaris samples. Higher percentages of Rhizobiaceae and Hyphomonadaceae families were observed in apical parts (Figure 2C and Supplementary Figure S7). In particular, *Nitratireductor* (Rhizobiaceae) and *Litorimonas* (Hyphomonadaceae) were key genera which contributed to 5% of the dissimilarity between the apical and basal parts (Supplementary Table S10). In contrast, the basal parts showed higher relative percentages of several families such as Saprospiraceae (Figure 2C), notably several discriminant genera such as *Lewinella* and *Portibacter* (Supplementary Figure S7 and Supplementary Table S10). Moreover, *Algitalea* was also observed as the main discriminant genus of the basal parts of Tamaris samples (Supplementary Figure S7) contributing to 4 and 2% of the dissimilarity with apical parts for Carqueiranne and Tamaris, respectively (Supplementary Table S10). Finally, it can be noticed that no sequences affiliated to Archaea were observed for algal samples in contrast with water and rocky samples for which the only detected OTU was below 0.06% of sequences and was affiliated to Nitrosopumilaceae (Thaumarchaeota).

**FIGURE 3:**
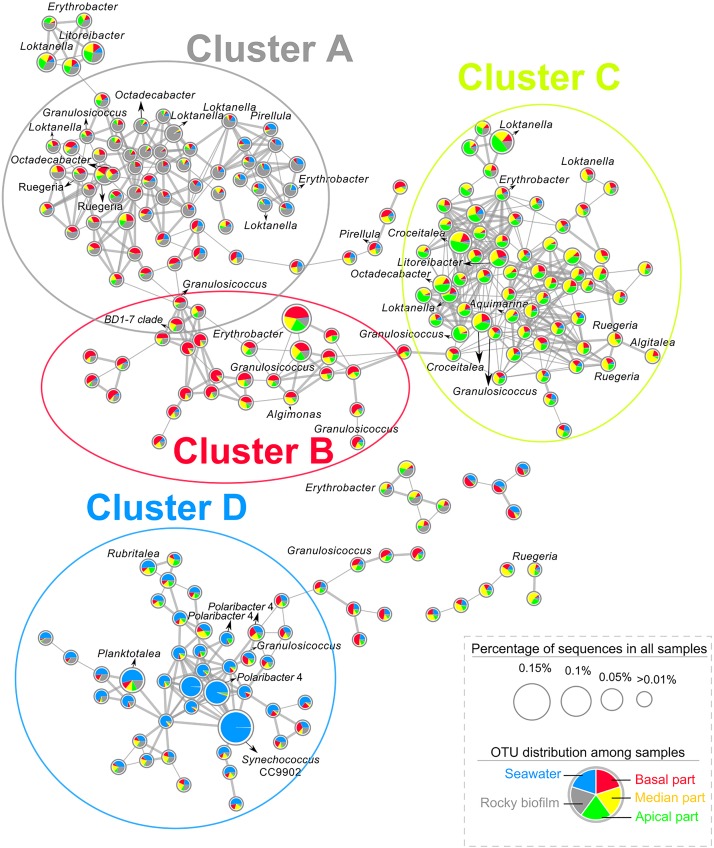
Co-occurrence network of OTUs from the 16S rDNA dataset of epibacterial communities at the surface of *T. atomaria*, in rocky biofilms and in seawater at Carqueiranne. Pie-chart inside each node revealed the distribution of each OTU across the different sample groups (Red: basal part, yellow: median part, green: apical part, gray: rocky biofilm, blue: seawater). Thickness of edges between each node was proportional to correlation or dissimilarity distances. The size of each node was proportional to the relative percentage of sequences of each OTU in all samples.

A co-occurrence network was built at the OTU level for each site using water, rocky and *T. atomaria* samples. For both networks, a high number of OTUs co-occurred in basal and median algal parts, and rocky biofilms. At Carqueiranne, a first cluster (cluster A, Figure 3) showed not only OTUs mainly present in rocky biofilms but also OTUs co-occurring in rocky biofilms and basal or median algal parts. These OTUs were mainly affiliated to the genera *Octadecabacter*, *Ruegeria*, *Loktanella*, and *Granulosicoccus*. Cluster A showed several links with cluster B which exhibited OTUs, mainly affiliated to *Erythrobacter*, *Granulosicoccus*, and *Algimonas*, with a high specificity for basal and median algal parts. Cluster C was composed by OTUs highly specific of median and apical parts: they were mainly affiliated to the genera *Loktanella*, *Ruegeria*, *Algitalea*, *Croceitalea*, and *Aquamarina*. Cluster D included OTUs mostly occurring in water samples, with the predominance of several genera such as *Synechococcus*, *Polaribacter*, and *Planktomarina*. For Tamaris samples, a similar clustering pattern was also observed (Supplementary Figure S9). In this network, no OTUs co-occurring mainly in apical algal parts and rocky biofilms were found. Cluster A was composed by OTUs mainly found in rocky biofilms or which co-occurred in rocky biofilms, and in water samples and/or on basal algal parts. Similarly, this cluster was connected to cluster B which showed OTUs mainly found on basal and median parts of the thalli. Cluster B was also connected to the cluster C which was composed of OTUs dominant in apical algal parts. Finally, cluster D displayed OTUs mainly found in water samples, with the predominance of the genera *Polaribacter* and *Planktomarina*.

Venn diagrams (Supplementary Figure S10) showed also a higher percentage of common OTUs between algal samples and rocky biofilms rather than between algal and water samples (Carqueiranne: 58.4% vs. 50.8%, respectively; Tamaris: 47.1% vs. 41.0%, respectively). The algal parts sharing the highest percentages of sequences with only rocky biofilms were the basal ones (Carqueiranne: 3.5%, Tamaris: 2.7%).

### Comparative Metabolomics Fingerprinting Analysis

After extraction and filtering of the raw chromatographic data obtained from all algal samples (total and surface extracts), three data matrices were obtained with 433, 135 and 261 *m/z* features for the LC-(+)-ESI-MS, LC-(−)-ESI-MS and GC-MS analyses, respectively. Chemodiversity estimation of surface extracts calculated with the Shannon index showed in some instances higher values at the apical parts (Supplementary Figure S11). More precisely, for LC-(+)-ESI-MS analyses of samples collected at Carqueiranne and for LC-(−)-ESI-MS and GC-MS analyses of samples from Tamaris, the resulting surface metabolomes from apical parts showed the highest chemodiversity. Furthermore, LC-(+)-ESI-MS was the analytical approach which revealed the larger chemodiversity in all sample groups.

**FIGURE 4:**
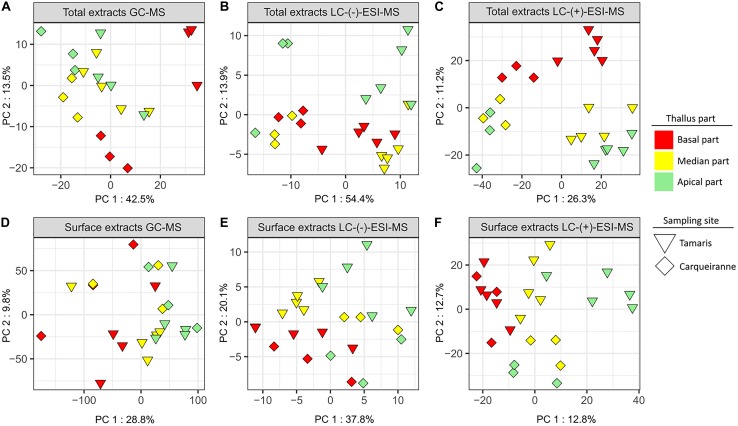
PCA plots of GC-MS **(A,D)**, LC-(−)-ESI-MS **(B,E)**, and LC-(+)-ESI-MS **(C,F)** metabolomics analyses of surface and total extracts of *T. atomaria* (two sites: Carqueiranne and Tamaris; three algal parts: basal, median, and apical).

For total extracts, the resulting PCA plots, but also to a higher extent PLS-DA plots, showed a significant discrimination according to the sampling sites on the first component, while samples were discriminated according to the thallus parts on the second component (Figures 4A–C, Supplementary Figures S12A–C, and Supplementary Table S11). The zonal discrimination was observed linearly from the basal to the median and then to the apical algal parts with all analyses (Figure 4 and Supplementary Figure S12), excepted for the PCA plot built with the LC-(−)-ESI-MS data (Figure 4B). This observation indicated a continuous global shift of the total metabolome composition along the thalli. For GC-MS and LC-(+)-ESI-MS analyses, pairwise tests revealed that the basal parts were significantly different from the apical and median ones (Supplementary Table S12).

For surface extracts, LC-(+)-ESI-MS analysis also showed a clear discrimination according to both sampling sites and zonal distribution, with a high proximity between the chemical composition of the basal parts from both sites (Figure 4F and Supplementary Figure S12F). In this case, pairwise tests also revealed that the basal parts were significantly different from the apical and median ones (Supplementary Tables S11, S12). In the case of LC-(−)-ESI-MS data, the discrimination between the samples appeared less evident but can still be observed between sites on the first component and, only in the case of algal samples from Tamaris, between thallus parts on the second component (Figure 4E and Supplementary Figure S12E). Significant differences were still observed with the pairwise analysis between both sites and between the algal parts (Supplementary Tables S11, S12). GC-MS analysis showed a higher variability between replicates and no discrimination can be clearly observed from multivariate plots (Figure 4D and Supplementary Figure S12D). In this case, only the apical parts were significantly different from the other parts (Supplementary Tables S11, S12).

Finally, it can be noted that for all PLS-DA analysis (except for surface extracts analyzed by GC-MS), cross validations strengthened these results showing differences between algal parts (*R*^2^ > 0.8 for all, Supplementary Table S13).

### Global Annotation of Metabolomes

The 25 major metabolites from the GC-MS dataset were attributed to sesquiterpenes. Among them, 17 were annotated by comparing their MS spectra and retention indexes with those found in databases (Supplementary Table S14). More specifically, gleenol was identified through the comparison of the experimental data with those obtained with a purified standard (Othmani et al., 2016b).

For the LC-MS datasets, a MS/MS molecular network was built for each mode (positive and negative) using the GNPS platform with both extract types. For the LC-(+)-ESI-MS/MS network (Supplementary Figure S13), clusters A and E gathered lipids including mainly diacylglycerylhydroxymethyl-*N*,*N*,*N*-trimethyl-β-alanines (DGTAs) and phosphatidylcholines (PCs), but also sulfoquinovosyldiacylglycerols (SQDGs), diacylglycerols (DGs), and monogalactosyl-diacylglycerols (MGDGs). For some of them, these lipid families were characterized through specific fragment ions, such as *m/z* 236.1494 [C10H22NO5]^+^ for DGTAs and *m/z* 184.0722 [C_5_H_15_NO_4_P]^+^ for PCs. DGTAs were distinguished from their structural isomers diacylgycerol-*N*-trimethylhomoserines (DGTSs) based on the absence of a characteristic loss of *m/z* 87 (Roche and Leblond, 2010), even if it is matter of controversy (Li et al., 2017). Clusters B and F consisted of terpenoids, including several sesquiterpenes and geranylgeranylglycerol (GGG) previously purified from *T. atomaria* (Othmani et al., 2016a, b). In cluster B, several compounds were putatively annotated as GGG derivatives on the basis of their MS/MS fragmentation pattern. Cluster C was constituted by *lyso*-DGTAs identified, as in the case of DGTAs, through the characteristic fragment ion at *m/z* 236.1494. Finally, Cluster D was found to contain pheophytin A and derivatives.

The LC-(−)-ESI-MS/MS-based molecular network (Supplementary Figure S14) mainly showed the occurrence of phospholipids and sulfolipids. Cluster A gathered phosphatidylethanolamines (PEs) annotated on the basis of their characteristic fragment ion at *m/z* 140.0119 [C_2_H_7_NO_4_P]^–^. A second cluster (cluster B) gathered sulfoquinovosyldiacylglycerols (SQDGs) and sulfoquinovosylmonoacylglycerols (SQMGs) elucidated with the help of the typical fragment ion at *m/z* 225.0070 [C_6_H_9_O_7_S]^–^.

Finally, by using molecular networking for both LC-ESI-MS/MS methods, a total of 82 lipids were putatively annotated including 32 DGTAs/*lyso*-DGTAs, 25 SQDG/SQMGs, 9 *lyso*-PEs, 7 DGs, 5 PCs/*lyso*-PC, 2 GGG derivatives, 2 MGDGs.

### Variations of Surface Metabolites Involved in the Differentiation Between Thallus Parts

**FIGURE 5:**
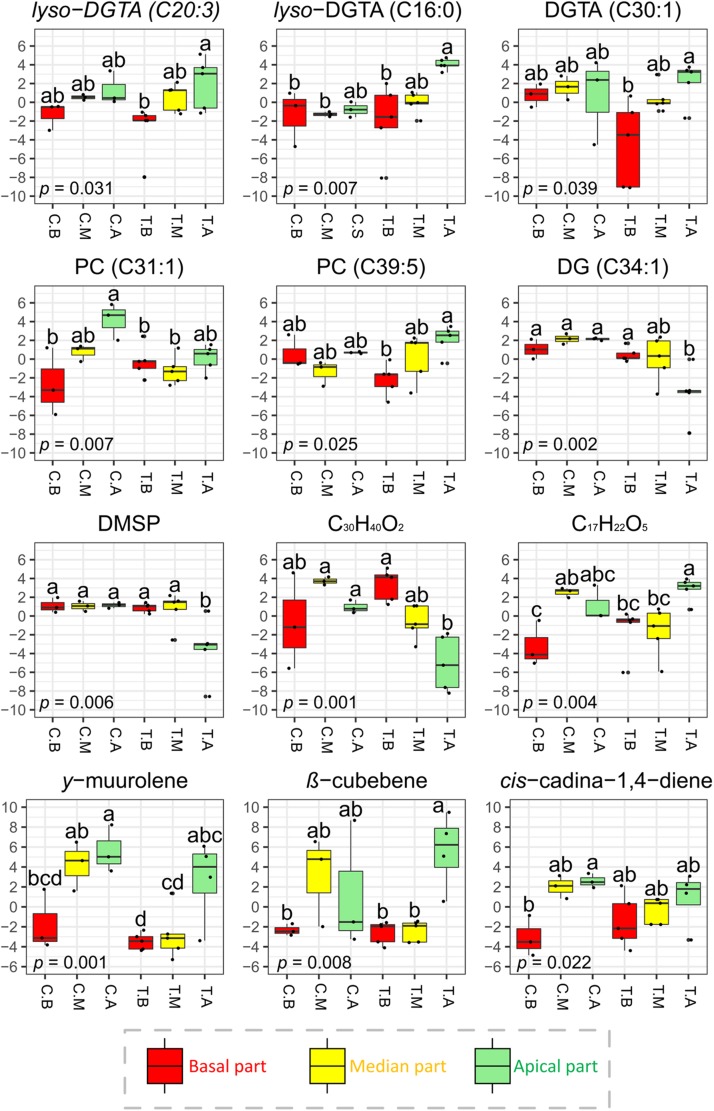
Normalized concentrations of selected surface metabolites (significant VIP features) detected by LC-(+)-ESI-MS and GC-MS across the different parts of the thalli of *T. atomaria*. Boxplots in red, yellow and green (labeled “B,” “M,” and “A”) represented basal, median, and apical algal parts, respectively. Boxplots corresponding to samples collected at Carqueiranne or Tamaris were labeled “C.” and “T.”, respectively. *P*-values and “a,” “b,” and “c” indexes correspond to the results of one-way ANOVA analyses and *post hoc* tests (HSD Tukey’s test), respectively.

Among the major compounds detected by GC-MS, sesquiterpenes were mainly found at the surface of the apical parts (Supplementary Figure S15). As example, *γ*-muurolene for both sites, *cis*-cadina-1,4-diene for Carqueiranne samples and β-cubebene for Tamaris samples, were predominantly found on the apical parts (Figure 5).

For the LC-(+)-ESI-MS analysis, attention was focused on several surface metabolites determined from the PLS-DA according to their statistical significance and VIP score. Among the 31 VIPs characterized in the resulting dataset, 6 were annotated as DGTAs (or *lyso*-DGTAs, Supplementary Tables S15, S16). In comparison to the basal parts, these lipids were found in higher amounts on the apical ones at both sites [e.g., *lyso*-DGTA (C20:3), *lyso*-DGTA (C16:0) and DGTA (C30:1) for Tamaris samples; *p* < 0.05] (Figure 5 and Supplementary Figure S16). No significant differences were observed with the *post hoc* test between the median parts and the two other algal parts. For Carqueiranne samples, among the 15 VIP features characterized, 5 were annotated as PCs through the analysis of their MS and MS/MS data (Supplementary Table S13). PCs were mainly produced on the apical parts [e.g., PC (C31:1) and PC (C39:5) for Carqueiranne and Tamaris samples, respectively; *p* < 0.05] (Figure 5 and Supplementary Figure S16). Unfortunately, several VIPs remained unidentified even if a chemical formula was proposed (Supplementary Tables S15, S16). Among them, two compounds (C_29_H_40_ and C_17_H_22_O_5_) were found in significant lower amounts on the basal parts in comparison to the median ones at Carqueiranne, and to the apical parts at Tamaris (Figure 5 and Supplementary Figure S16). In contrast, only few metabolites were found in lower quantities at the apical parts, including for Tamaris, a diacylglycerol [DG (C34:1)], DMSP, and a putative *apo*-carotenoid (C_30_H_40_O_2_) (*p* < 0.05, Figure 5). For the LC-(−)-ESI-MS method, neither PEs, SQMGs, nor SQDGs identified in the dataset (Supplementary Figure S17) were found among the most discriminant (VIP scores < 2) and significant features.

Finally, while the majority of PCs, DGTAs and sesquiterpenes were found in significant higher amounts at the apical parts of surface extracts, the same trends were not significantly observed, for their majority, in the case of total extracts (Supplementary Figures S15, S16).

### Integration of Surface Metabolome and Surface Microbiota Datasets

**FIGURE 6:**
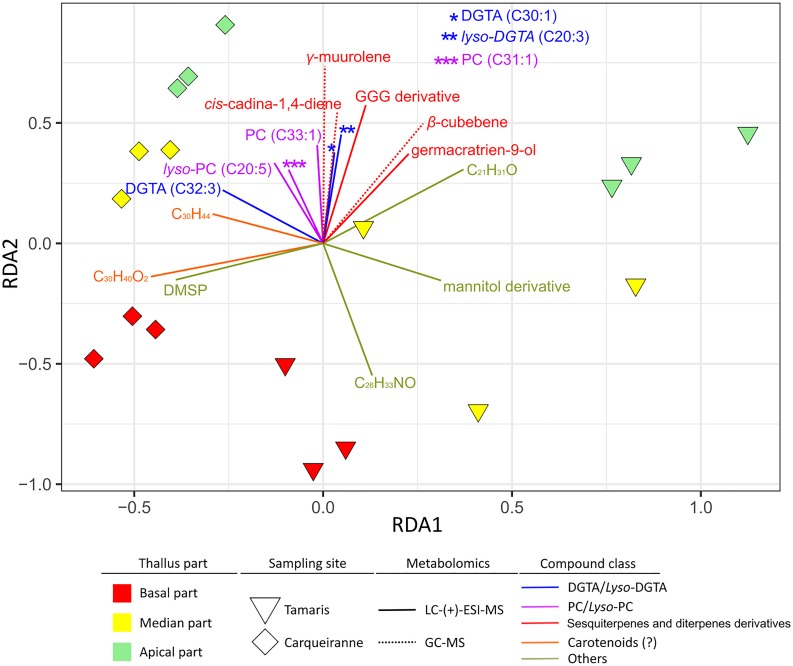
Weighted UniFrac distance-based redundancy analysis (db-RDA) plot constructed with the 16S rDNA dataset of epibacterial communities at the surface of *T. atomaria* and significant surface metabolites (VIPs) as explaining variables.

A distance-based redundancy analysis (db-RDA) was performed using weighted UniFrac distance matrix from 16S rRNA gene sequencing dataset and normalized concentrations of surface metabolites of interest as explaining variables. Surface compounds were selected according to their statistical significance and VIP score (>2). Nevertheless, in some cases, redundancy was observed between metabolites of the same chemical family (e.g., DGTAs, PCs or sesquiterpenes). Thus, only a limited number of compounds were selected and considered as representative of their respective chemical class (Figure 6, permutation test: *p* = 0.042).

Several groups of metabolites, discriminated on the second RDA axis, could be viewed as factors directly or indirectly correlated with specific microbiota from the apical parts whatever the site. Among these metabolites, some (*lyso*-)DGTAs and (*lyso*-)PCs but also several sesquiterpenes, such as β-cubebene, γ-muurolene, *cis*-cadina-1,4-diene, and germacratrien-9-ol, appeared as potential specific drivers of the apical microbiota. Only an unidentified compound (C_26_H_33_NO) seemed to be more specifically linked with basal communities of Tamaris samples. While DMSP, putative *apo*-carotenoids (C_30_H_40_O_2_ and C_30_H_44_) and a putative mannitol derivative, appeared discriminant along thalli from Tamaris (Figure 5), these metabolites were also involved in the chemical differentiation between both sites.

## Discussion

Seaweed surface microbiota is increasingly studied in the holobiont context but thalli are generally considered as a whole. However, we assumed that a physiological differentiation occurred along the thallus of *T. atomaria* since meristematic cells are located in the apical part which is consequently the zone where growth happens (Robinson, 1932). Thus, one specific aim of this study was to investigate if the growth of the alga could influence the surface-associated microbiota at the thallus scale.

### Specific Microbiota Selected at the Algal Surface

The algal epibacterial community structure was compared to communities from seawater and rocky biofilms to assess the host-specificity. Similarly to Canadian kelps and *Mastocarpus* spp. (Lemay et al., 2018a, b), a clear discrimination was observed between overall algal, planktonic, and rocky communities. Dissimilarities between seawater and biofilms underlined the specificity of the ecological niche that represents the life at a surface (Flemming et al., 2016), including algal surface (Lemay et al., 2018a; Sneed et al., 2015). The specificity of algal surface microbiota with respect to that found on rocky biofilms in similar environmental conditions strengthens the hypothesis of an active role of the algal host in the selection of epiphytic communities. Moreover, the main bacterial taxon involved in the dissimilarity with rocky biofilms, *Algitalea*, has been previously described from the surface of the Chlorophyta *Ulva pertusa*, as a new genus (Yoon et al., 2015). It can be noticed that the specificity is all the higher as rocks sampled were collected at both sites and corresponded to diversified mineral substrates with various physico-chemical properties (e.g., roughness) and probably various times of immersion. Interestingly, no Archaea were identified for algal samples when rocky biofilms exhibited low relative abundances (<0.1%), in the same range as for biofilms formed on artificial surfaces (Pollet et al., 2018; Catão et al., 2019).

### Epibacterial Communities Differed From the Basal to the Apical Algal Parts

Epibacterial cells were found in significant higher densities at the basal parts of *T. atomaria* collected at Tamaris. A similar tendency has been previously observed at the surface of *Ulva australis* and *Delisea pulchra* with densities from 10^6^ cell.cm^–1^ at the distal parts to more than 10^7^ cells.cm^–1^ at the basal parts (Tujula, 2006; Maximilien et al., 1998). In the case of *T. atomaria*, a lower order of magnitude was observed, ranging from 10^5^ to 10^6^ cell.cm^–1^. Such inter-species differences could be explained by the host-specificity or local environmental conditions.

We tried to visualize surface microbiota of algal parts through a CLSM approach. Images revealed the presence of diatoms, but also probably of fungi hyphae or heterotrophic filamentous bacteria. When comparing this information with the 16S rRNA gene metabarcoding dataset, the major taxa of heterotrophic filamentous bacteria found were Thiothricaceae, and more particularly *Leucothrix* spp., which have been already reported as seaweed epiphytes (Bland and Brock, 1973).

α*-*Diversity metrics decreased from the basal to the apical parts of *T. atomaria* at both sites. Similar results have been previously reported for the Phaeophyceae *Sargassum muticum* for which the α-diversity of the bacterial communities is significantly higher at the holdfast in comparison to the tips (Serebryakova et al., 2018). By contrast, no significant differences have been determined for *Fucus vesiculosus* when comparing tips, thallus and the whole seaweed (Parrot et al., 2019).

β-Diversity analyses revealed both site and “thallus-part” specificities of the community structure, which suggested that microbiota structure differed significantly depending on the geographical area but also, at a smaller scale, along the thallus. Even if no environmental characterization was performed for this study, Tamaris is located within the Toulon bay, and exhibited a higher level of anthropization compared to Carqueiranne (e.g., trace metals, Coclet et al., 2019). A more thorough study should be carried out to test if environmental parameters could explain the differences observed between both sites, whatever the thallus part.

Along thalli from both sites, two Bacteroidetes families constituted key taxa of surface associated communities with global constant proportions. However, some unknown Saprospiraceae genera were mainly associated to the basal parts at both sites whereas Flavobacteriaceae genera (*Algitalea*, *Croceitalea*, and *Winogradskyella*) belonged to the pioneer communities found on the apical parts of *Taonia*. At the apical parts of samples from Tamaris, a higher percentage of Hyphomonadaceae, and more particularly the genus *Litorimonas*, was observed in comparison to other algal parts. In the case of *Fucus vesiculosus*, the family Hyphomonadaceae has been also observed with a higher relative abundance on the tips (Parrot et al., 2019). Hyphomonadaceae have been also reported to produce a polar holdfast structure which facilitates surface colonization (Dang and Lovell, 2016). Therefore, Hyphomonadaceae could represent a pioneer family able to colonize the youngest parts of the thalli. This result was strengthened by observations we have made in a previous temporal study when Hyphomonadaceae have been observed as one of the main family in February and March, the early period of occurrence of *T. atomaria* in the French Mediterranean coasts (Paix et al., 2019).

The similarity of structure with rocky biofilms, which were not the substrates where algal thalli were settled, decreased from the basal to the apical parts. However, rocky biofilms probably included communities with dissimilar ages and surface natures which prevent to go further on if the relative specificity of the different parts of the thalli differs. The higher abundance and α-diversity at basal compared to apical zones could be explained by the maturation of the biofilm (potentially including EPS production). In addition, maturation was reputed to improve the ability of biofilms to limit the influence of environmental pressures (Flemming et al., 2016) which could explain the higher similarity of the basal communities at both sites.

### Cross Metabolomics and Molecular Networking Allowed to Attribute to Algal Metabolites the Intra-thallus Variations of the Surface Metabolome

A multiple platform metabolomics approach combining LC-(+)-ESI-MS, LC-(−)-ESI-MS and GC-MS analyses offered a comprehensive coverage of the total and surface metabolomes of *T. atomaria*. While both LC-ESI-MS methods gave a good coverage of the lipidome, the complementary GC-MS approach allowed a better view of the surface “volatilome” of the seaweed since a higher range of sesquiterpenes were identified. Moreover, the ability of the dipping method used here to extract a large part of the surface metabolome of *T. atomaria* was validated by the fact that during this study, but also in a previous work (Paix et al., 2019), polar compounds (e.g., DMSP, mannitol or dipeptides) have been also detected in addition to lipophilic compounds more logically expected (e.g., fatty acid derivatives and terpenes). To date, only few studies have been used to decipher the chemical production of macroalgae by metabolomics. Unfortunately, in most of them, only a few number of metabolites were identified.

The annotation of large metabolomics datasets is often considered as the most challenging step in metabolomics studies. The recent development of molecular networking as an annotation tool allowed a more powerful identification of metabolites for non-model organisms (Wang et al., 2016). In the specific case of macroalgae which are known to produce a wide range of lipids, such an analytical tool seems particularly adapted as these compounds show similar MS/MS fragmentation pattern and could be thus gathered in clusters facilitating their annotation (Parrot et al., 2019; Paix et al., 2019).

In the context of this study, the main families of surface compounds implied in the intra-thallus variations were DGTAs, terpenes and PCs (Supplementary Tables S14, S15). Among these compounds, DGTAs are known to be mainly produced by eukaryotic organisms, and more particularly by Ochrophyta, while the occurrence of DGTAs in bacteria has been only scarcely reported in the literature (Dembitsky, 1996; López-Lara et al., 2003). Concerning terpenes found at the algal surface, the main part of them have been already isolated in relatively high amounts from the total extracts of *T. atomaria* (Othmani et al., 2016b) and are typical of the family Dictyotaceae (e.g., cyclic sesquiterpenes and GGG). Conversely, few of these compounds have been described to date from prokaryotes. The case of PCs was more controversial because, even if such compounds are considered as one of the main membrane phospholipids in eukaryotes, it has been shown recently that some bacteria can also biosynthesize PCs (Sohlenkamp and Geiger, 2016). Moreover, typical bacterial membrane lipids such as PEs, phosphatidylglycerols (PGs) or the more specific ornithine lipids (OLs) were not detected and/or not implied in the intra-thallus chemical discrimination of the surface extracts. All these reasons led us to believe that the chemical production of the algal host was predominant in the surface metabolome and that, in the case of *T. atomaria*, it was heavily involved in the differences observed at the thallus scale. Nevertheless, we are also aware of the limitation of our method because it did not allow us to characterize all the components of the phycosphere, in particular higher-molecular-weight molecules such as polysaccharides, proteins or nucleic acids.

### Algal Growth Could Explain Zonal Variations of Metabolome and Microbiota

With a clear discrimination between sites and thallus parts, the surface metabolomes [more particularly those analyzed by LC-(+)-ESI-MS] showed a similar clustering pattern than that observed for the microbial β-diversity. As it has been already reported in the case of temporal variations of the same holobiont model (Paix et al., 2019), this similar trend suggested that a high number of co-variations occurred between surface metabolites and epibacterial OTUs.

Nevertheless, while the α-diversity of prokaryotic communities decreased from the basal to the apical parts, the opposite tendency was observed for the chemodiversity of surface compounds. It appeared that surface of the apical parts is composed by a more diverse range of chemical families than the older algal parts (base and median parts).

Annotation allowed to confirm that trend since higher amounts of PCs, DGTAs and sesquiterpenes were specifically found on apical parts. Similarly, DGTAs (and/or DGTS) have been also identified as the main components of the surface lipidome of *F. vesiculosus*, one of these betaine lipids being also found in higher concentrations in the upper part of the thallus (Parrot et al., 2019). DGTAs have been previously found to be mainly expressed during the early period of occurrence of *T. atomaria* (February and March; Paix et al., 2019). These results could indicate that these betaine lipids are produced in higher relative concentrations at the membrane of cells during their early stage. Therefore, among factors which could explain the difference of metabolome at the thallus scale, growth of the seaweed must be taken into account. In a recent study, the total content of DGTAs of the Ochrophyta *Sargassum honeri* has been found to first increase during the early growth phase of the sporophytes, and then to decrease later when the alga reaches its mature stage (Zhang et al., 2018). Moreover, the lipid content of the Rhodophyta *Porphyra dioica* has been investigated and specific profiles of betaine lipids and phospholipids have been reported depending of the life stages (gametophytes or sporophytes), indicating the importance of the variations of the membrane lipidome during the whole life cycle of the seaweed (da Costa et al., 2018).

In the case of *T. atomaria*, it has been shown that growth takes place at the level of meristems which are located at the apex (Robinson, 1932). Thus, cell membranes from the apical part of the seaweed could be characterized by a chemical composition specific to the early stages of growth, including potentially PCs and DGTAs. This specificity of apical membranes could induce the selection of specific epibacterial communities and could be a realistic assumption to explain why the epibacterial community differed significantly by its abundance and/or structure along the thallus.

The presence of reproductive structures could also play a key role in the zonal profiling of the surface metabolome. The development of *T. atomaria* from British coasts has been previously studied and characterized by the production of “hairs bands” and tetrasporangia at the apical parts of the thallus (Robinson, 1932). These reproductive structures were also reported in this study at the surface of the apical parts and, in less cases, on the median parts (data no shown). This observation could also explain the metabolome differences reported along the thallus, with specific families of compounds produced in higher concentrations at the apical parts. The presence of “hairs bands” and tetrasporangia at apical parts could also modify the size and the morphology of the surface at a micro-scale and thus could directly impact the adhesion efficiency of specific microbial communities. Thus, differentiated textures at the algal surface, as reported for aquatic plants (Yang et al., 2013), could be involved in such a dissimilar colonization.

In the case of the seaweed *Mastocarpus* spp., a recent study has also highlighted the importance of the host life-cycle on microbial associated communities (Lemay et al., 2018a). In connection with our study, the sexual and even the growth state of the algal host could be considered as key factors when studying seaweed microbial communities.

Moreover, the meristematic growth of the alga implies that the apical parts are younger than the rest of the thallus. Conversely, the basal zones constitute the first parts of the young sporophytes and then would be subjected to a longer period of exposure to planktonic microbial communities. The basal zones consequently experienced microbial communities’ succession together with biofilm maturation including EPS secretion, which could explain a higher microbial density and diversity. Moreover, especially for mature thalli, the apical parts suffer from higher amplitudes of movement in the seawater and consequently shear stress could perhaps also impact the colonization process at their surface.

### Specific Role of Algal Secondary Metabolites on Epibacterial Communities

Dimethylsulfoniopropionate has been already studied in the case of several other seaweed-holobiont models and showed interesting key ecological roles involved in the bacterial-seaweed interactions such as anti-adhesion properties for *Fucus vesiculosus* (Saha et al., 2011, 2012) or microbial gardening for the morphogenesis of *Ulva mutabilis* (Wichard and Beemelmanns, 2018). Additionally, DMSP has also showed other functions such as cryoprotection, defense against herbivory, and antioxidant properties (Karsten et al., 1992; Sunda et al., 2002; Lyons et al., 2010). In this study, DMSP was found in lower concentrations at the apical parts in comparison of basal ones for samples collected at Tamaris. Previously, DMSP has been reported in significant lower amounts at the surface of *T. atomaria* during the winter period (early period of occurrence on the sampling site) compared to the spring and summer periods (Paix et al., 2019). Considering that the apical parts are younger than the basal ones, DMSP could be viewed as a metabolite expressed by the alga after the early period of occurrence of the sporophytes or produced by some microorganisms (e.g., diatoms) found in mature biofilms. However, clear causalities links remain difficult to assume, and the role of this metabolite needs to be more specifically investigated in *T. atomaria*. Besides, several bacterial taxa from the *Roseobacter* clade (Rhodobacteraceae) are well known for their catabolism associated to DMSP with several enzymes, such as DddD and DddP, involved in the cleavage of DMSP into dimethyl sufide (DMS) (Curson et al., 2011). A focus on these enzymatic pathways, together with a precise quantification of DMSP at the algal surface would constitute a complementary perspective to allow further hypothesis on the role of this compound on the zonal specificity of the epibiota of *T. atomaria*.

In Othmani et al. (2016a), two sesquiterpenes (gleenol and *trans*-calamenene) have been described for their anti-adhesion activities against several bacterial strains at relevant natural concentrations. In the present work, all sesquiterpenes detected, including gleenol and *trans*-calamenene, were detected with increasing concentrations from the basal to the apical algal parts. Sesquiterpenes are well-known, in the case of terrestrial plants, as volatile organic compounds particularly present in reproductive organs and playing a role in the plant growth, but also in their chemical defense against microbial pathogens (Junker and Tholl, 2013; Boachon et al., 2019). More broadly, the optimal defense theory assumes that the level of defenses varies among organism parts as the resources are allocated in order to have the best benefit/cost ratio in terms of fitness (McKey, 1979). In that way, higher concentrations of defense sesquiterpenes could be specifically produced at the surface of the apical parts which are the more valuable for the algal fitness, and thus these compounds could be involved in the selection of specific epibacterial communities. Since the apical parts are constituted by meristematic cells, together with reproductive structures (tetrasporangia and “hair bands”), sesquiterpenes could be exuded at the surface as defense compounds to protect these specific types of differentiated cells.

## Conclusion

Only few studies were focused on microbiota variations at the scale of a single algal individual. Thus, factors explaining the variations of epiphytic microbiota still need to be more investigated to better understand the ecology and physiology of seaweed holobionts. This study is the first one which confirmed that surface microbiota of a seaweed displayed host-specificity and differed significantly and gradually from the basal to the apical parts in abundance, α-diversity and structure in relation with a similarly differentiated surface metabolome. Based on these main results, this study suggested a plausible scenario where the algal physiology along the thallus surface could explain such variations. In addition, with a complementary metabolomics approach, the meristematic growth was proposed to involve several lipids, such as DGTAs and PCs, but also chemical defenses with several sesquiterpenes acting in the selection of microbial communities.

## Data Availability Statement

Sequences data were deposited and are publicly available in the NCBI Sequences Read Archive (SRA) under the BioProject ID PRJNA553652, accession number. Raw data for LC-ESI-(+)-MS/MS and LC-ESI-(−)-MS/MS experiments were deposited and are publicly available in the MassIVE platform under the IDs MSV00008337 and MSV000083084, respectively. GC-MS and cytometry raw data are available on request from the authors.

## Author Contributions

BP, J-FB, and GC designed the study and wrote the manuscript. BP, RB-M, SG, BM, J-FB, and GC performed the experiments and data acquisition. BP, NC, RB-M, and SG contributed to the treatment of the raw data. BP, NC, J-FB, and GC contributed to the data analysis.

## Conflict of Interest

The authors declare that the research was conducted in the absence of any commercial or financial relationships that could be construed as a potential conflict of interest.

## Footnotes

1 http://bioinformatics.psb.ugent.be/webtools/Venn/

2 https://workflow4metabolomics.org/

3 https://www.metaboanalyst.ca/

4 https://gnps.ucsd.edu/
