# Supplementary material for: A Multi-Omics Analysis Suggests Links Between the Differentiated Surface Metabolome and Epiphytic Microbiota Along the Thallus of a Mediterranean Seaweed Holobiont
Source: Front Microbiol. 2020 Mar 25;11:494. doi: 10.3389/fmicb.2020.00494 (PMC7111306; doi:10.3389/fmicb.2020.00494)
Supplement: Supplementary file 1 [file Data_Sheet_1.docx]

**Supplementary information**

# A multi-omics analysis suggests links between the differentiated surface metabolome and epiphytic microbiota along the thallus of a Mediterranean seaweed holobiont

Benoit Paix^1^, Nathan Carriot^1^, Raphaëlle Barry-Martinet^1^, Stéphane Greff^2^, Benjamin Misson^3^, Jean-François Briand^1,*^ and Gérald Culioli^1,*^

^1^ Université de Toulon, MAPIEM, EA 4323, Toulon, France.

^2^ CNRS, Aix Marseille Université, IRD, Avignon Université, Institut Méditerranéen de Biodiversité et d’Ecologie marine et continentale. Station marine d’Endoume, Marseille, France.

^3^ Université de Toulon, Aix Marseille Université, CNRS, IRD, Mediterranean Institute of Oceanography (MIO), UM110, France.

^*^Corresponding authors: J.-F. Briand ([briand@univ-tln.fr](mailto:briand@univ-tln.fr)) and G. Culioli ([culioli@univ-tln.fr](file:///C:\Users\culioli\Desktop\culioli@univ-tln.fr))

# Supplementary Materials & Methods

## Supplementary information for LC-ESI-MS analyses

The UPLC-HRMS instrumentation consisted of a Dionex Ultimate 3000 Rapid Separation (Thermo Fisher Scientific, Waltham, MA, USA) chromatographic system equipped with a RS pump, a temperature-controlled autosampler, a thermostated column compartment and an UV-vis diode array detector. This system was coupled to a QToF Impact II mass spectrometer (Bruker Daltonics, Bremen, Germany). The analyses were performed using an analytical core-shell reversed-phase column (150 × 2.1 mm, 1.7 *μ*m, Kinetex Phenyl-Hexyl equipped with a SecurityGuard cartridge, Phenomenex, Le Pecq, France) with a column temperature of 40°C and a flow rate of 0.5 mL.min^-1^. The autosampler temperature was set at 4°C and the injection volume was 5 *μ*L. Mobile phases were: (A) water and (B) acetonitrile (Chromasolv; Sigma-Aldrich-Merck, Darmstadt, Germany) containing each 0.1% (*v*/*v*) of formic acid (Ultra grade; Fluka, Fischer Scientific, Illkirch, France). The elution gradient started at 5% B and maintained for 2 min, then increased to 100% B (linear ramp) in 8 min and maintained for 4 min; then back to 5% B (linear ramp) over 0.01 min and maintained 1.99 min, for a total run time of 16 min.

The capillary voltage of the MS spectrometer was set at 4500V (positive mode), and the nebulizing parameters were set as follows: nebulizing gas (N_2_) pressure at 0.4 bar, drying gas (N_2_) flow at 4 L.min^-1^, and drying temperature at 180°C. Mass spectra were recorded from *m/z* 50 to 1200 at a mass resolving power of 25 000 full width at half-maximum (FWHM, *m/z* = 200) and a frequency of 2 Hz. Tandem mass spectrometry analyses were performed thanks to a collision induced dissociation (CID) with a collision energy of 25 eV. A solution of formate/acetate forming clusters was automatically injected before each sample for internal mass calibration, and the mass spectrometer was calibrated with the same solution before each sequence of samples. Data handling was done using DataAnalysis software (version 4.3, Bruker Daltonics).

## Supplementary information for GC-MS analyses

Separation of metabolites was performed on a HP-5MS 5% Phenyl-Methyl Silox column (30 m × 0.25 mm, 0.25 *μ*m; Agilent Technologies, Santa Clara, CA, USA) with helium as mobile phase. The run started at 40°C for 5 min and increased by 10°C.min^-1^ up to 350°C for a total runtime of 31 min. A constant flow rate was set to 1 mL min^-1^. A volume of 1 *μ*L of each sample was injected in splitless mode and the injector temperature was set at 250°C. A solution with a mix of C_8_-C_20_ alkanes (Fluka) was also injected for the determination of retention indices.

## Supplementary information for annotation procedure

In this study, three levels of annotation were applied for the identification of metabolites, following the same methodology described in Paix et al., 2019.

The first approach, which corresponds to the level 1 of annotation according to Schymanski et al., 2014, was based on the use of several commercial standards described in Paix et al., 2019, as well as compounds previously purified from *T. atomaria* or *Dictyota* spp. by our team (Othmani et al., 2016b; Viano et al., 2009). These standards were solubilized in MeOH at a concentration of 0.1 mg.mL^-1^ and analyzed with the same experimental conditions used for the three metabolomics workflows.

The second strategy was to annotate putatively some *m/z* features by the comparison of their MS (and MS/MS for LC-MS analyses) data with reference databases. A match found with a MS (and MS/MS) library is considered with the level 2 of annotation. To facilitate the dereplication procedure with the two LC-MS datasets, the molecular networking approach was used, allowing the clustering of metabolites with a similar chemical structure in a same sub-network (cluster). Briefly, molecular networks were generated using GNPS platform with surface and total extracts (<https://gnps.ucsd.edu/ProteoSAFe/static/gnps-splash.jsp>, Wang et al., 2016). MS/MS raw data (.mzxml files) were clustered using MS cluster with a tolerance of 0.02 Da for precursor ions and 0.02 Da for MS/MS fragment ions. Minimum cosine score (CS) value used for the clustering was set to 0.7. Data were exported and analyzed using Cytoscape (v. 3.4.0). Positive and negative LC-ESI-MS/MS molecular networks were then analyzed and annotated based on MS/MS fragmentation pathways and comparison with in-house and public databases such as Metlin (<https://metlin.scripps.edu/>) or Lipidmaps (<https://www.lipidmaps.org/>).

When no characteristic fragmentation pathway was determined by comparison to the literature, the most probable chemical formula was proposed using the *Smart Formula* tool from DataAnalysis. This last step finally corresponded to the level 4 of annotation.

**References**

Othmani, A., Bunet, R., Bonnefont, J.-L., Briand, J.-F., & Culioli, G. (2016). Settlement inhibition of marine biofilm bacteria and barnacle larvae by compounds isolated from the Mediterranean brown alga *Taonia atomaria*. *Journal of Applied Phycology*, *28*(3), 1975–1986. doi: 10.1007/s10811-015-0668-4

Paix, B., Othmani, A., Debroas, D., Culioli, G., & Briand, J.-F. (2019). Temporal covariation of epibacterial community and surface metabolome in the Mediterranean seaweed holobiont *Taonia atomaria*. *Environmental Microbiology*, in press. doi: 10.1111/1462-2920.14617

Schymanski, E. L., Jeon, J., Gulde, R., Fenner, K., Ruff, M., Singer, H. P., & Hollender, J. (2014). Identifying Small Molecules via High Resolution Mass Spectrometry: Communicating Confidence. *Environmental Science & Technology*, *48*(4), 2097–2098. doi: 10.1021/es5002105

Viano, Y., Bonhomme, D., Camps, M., Briand, J.-F., Ortalo-Magné, A., Blache, Y., … Culioli, G. (2009). Diterpenoids from the Mediterranean brown alga *Dictyota* sp. evaluated as antifouling substances against a marine bacterial biofilm. *Journal of Natural Products*, *72*(7), 1299–1304. doi: 10.1021/np900102f

# Supplementary Tables

## Table S1. Parameters used for metabolomics data processing (part 1)

| **XCMS 3.0 parameters used for LC-ESI-MS data processing** | Selected parameters |
| --- | --- |
| **Extraction method for peaks detection** | CentWave |
| Max tolerated ppm *m/z* deviation in consecutive scans in ppm | 2 |
| Min,Max peak width in seconds | 2,20 |
| Signal to Noise ratio cutoff | 10 |
| Prefilter step for for the first analysis step (ROI detection) | 0,0 |
| **Method to use for first grouping** | PeakDensity |
| Bandwidth | 10* / 30** |
| Minimum fraction of samples | 0.5 |
| Minimum number of samples | 1 |
| Width of overlapping *m/z* slices | 0.25 |
| Maximum number of groups to identify in a single *m/z* slice | 50 |
| **Method to use for retention time correction** | Obiwarp |
| Bin size (in *m/z* dimension) to be used for the profile matrix generation | 1 |
| **Method to use for second grouping** | PeakDensity |
| Bandwidth | 10 |
| Minimum fraction of samples | 0.5 |
| Minimum number of samples | 1 |
| Width of overlapping *m/z* slices | 0.25 |
| PeakDensityAdv |  |
| Maximum number of groups to identify in a single *m/z* slice | 50 |
| **Fill peaking** |  |
| Value by which the *m/z* width of peaks should be expanded | 0 |
| Value by which the RT width of peaks should be expanded | 0 |
| Specifying a ppm by which the *m/z* width of the peak region should be expanded | 0 |

* Value used for LC-(+)-ESI-MS dataset ; ** Value used for LC-(-)-ESI-MS dataset

## Table S1. Parameters used for metabolomics data processing (part 2)

| **Erah parameters used for GC-MS data processing** | Selected parameters |
| --- | --- |
| **Peaks detection and deconvolution** | |
| Minimum peak width | 2.5 |
| Minimum peak height | 2500 |
| Noise threshold | 500 |
| *m/z* to exclude | c(73:75,147:149,207:208) |
| **Alignment** | |
| Minimum correlation between spectra | 0.90 |
| Maximal time of misalignment | 20 |
| *m/z* range | 40:500 |
| **Missing compound recovery** | |
| Minimum number of samples | 3 |

## Table S2. Surfaces of the thallus parts used for metabolomics and sum of the chromatographic peak areas (surface extracts, LC-(+)-ESI-MS)

| **Site** | **Thallus part** | **Replicate** | **Surface of the thallus part (cm^2^)** | **Sum of the chromatographic peak areas** |
| --- | --- | --- | --- | --- |
| Carqueiranne | Basal | R1 | 0.86 | 68 637 650 |
|  |  | R2 | 1.00 | 78 057 335 |
|  |  | R3 | 1.01 | 82 430 445 |
|  | Median | R1 | 1.66 | 81 888 073 |
|  |  | R2 | 1.55 | 89 157 091 |
|  |  | R3 | 2.42 | 98 612 317 |
|  | Apical | R1 | 1.90 | 82 826 045 |
|  |  | R2 | 3.10 | 103 448 844 |
|  |  | R3 | 3.40 | 117 397 130 |
| Tamaris | Basal | R1 | 6.37 | 186 913 438 |
|  |  | R2 | 2.41 | 113 964 760 |
|  |  | R3 | 3.89 | 128 462 240 |
|  |  | R4 | 2.55 | 122 817 686 |
|  |  | R5 | 2.79 | 115 003 896 |
|  | Median | R1 | 5.92 | 183 219 063 |
|  |  | R2 | 9.62 | 201 502 962 |
|  |  | R3 | 6.12 | 194 114 469 |
|  |  | R4 | 5.22 | 147 675 204 |
|  |  | R5 | 2.72 | 111 884 657 |
|  | Apical | R1 | 6.02 | 157 565 425 |
|  |  | R2 | 10.27 | 230 346 718 |
|  |  | R3 | 4.28 | 153 539 839 |
|  |  | R4 | 3.64 | 118 921 859 |
|  |  | R5 | 8.14 | 182 534 138 |

## Table S3. Summary of PERMANOVA results examining the effect of “site” and “sample types” factors for of the 16S rRNA gene dataset.

D. f; MS; F; R^2^ and P correspond to degrees of freedom; mean square; F ratio; coefficient of determination and *p* value, respectively

|  | **D. f** | **MS** | **F** | **R^2^** | **P** |
| --- | --- | --- | --- | --- | --- |
| **Site** | 1 | 0.44573 | 6.5772 | 0.10667 | 0.001 |
| **Sample type** | 4 | 0.4745 | 7.0017 | 0.4542 | 0.001 |
| **Site:Sample type** | 4 | 0.11992 | 1.7695 | 0.11479 | 0.012 |
| **Residuals** | 20 | 0.06777 | 0.32435 |  |  |
| **Total** | 29 |  |  | 1 |  |

## Table S4. Multivariate pairwise results (*p* values) examining differences between both sites and between each sample types, for of the 16S rRNA gene dataset.

| **Comparison type** | **Samples compared** | ***p* values** |
| --- | --- | --- |
| **Site comparison** | Carqueiranne vs Tamaris | 0.002853067 |
| **Sample type comparison** | Basal vs median parts | 0.12997305 |
|  | Basal vs apical parts | 0.01248217 |
|  | Basal part vs seawater | 0.00815162 |
|  | Basal part vs rocky surfaces | 0.00815162 |
|  | Median vs apical parts | 0.31954351 |
|  | Median parts vs seawater | 0.00815162 |
|  | Median parts vs rocky surfaces | 0.00815162 |
|  | Apical parts vs seawater | 0.00815162 |
|  | Apical parts vs rocky surfaces | 0.00815162 |
|  | Rocky surfaces vs seawater | 0.00815162 |

## Table S5. SIMPER results of the most contributing genera to the dissimilarities between water samples and algal samples collected at Carqueiranne. Only the first 50% of the cumulative contribution is showed. *p* values were calculated with a permutation test constructed with 999 permutations.

| **Order** | **Family** | **Genus** | **Average contribution** | **SD** | **Ratio** | **Av. water samples** | **Av. thallus samples** | **Cum. sum** | ***p* value** |
| --- | --- | --- | --- | --- | --- | --- | --- | --- | --- |
| Synechococcales | Cyanobiaceae | *Synechococcus CC9902* | 10.9% | 0.10 | 1.09 | 1293 | 1 | 13.9% | 0.008 |
| Flavobacteriales | Flavobacteriaceae | *Algitalea* | 5.1% | 0.03 | 1.86 | 1 | 609 | 20.4% | 0.009 |
| Flavobacteriales | Flavobacteriaceae | *Polaribacter* | 4.4% | 0.06 | 0.75 | 543 | 38 | 26.0% | 0.034 |
| Chitinophagales | Saprospiraceae | unknown genus | 3.3% | 0.01 | 4.15 | 46 | 435 | 30.1% | 0.020 |
| Rhodobacterales | Rhodobacteraceae | *Litorimicrobium* | 2.9% | 0.02 | 1.65 | 357 | 8 | 33.9% | 0.002 |
| Pirellulales | Pirellulaceae | unknown genus | 2.7% | 0.02 | 1.52 | 324 | 0 | 37.3% | 0.018 |
| Chitinophagales | Saprospiraceae | *Lewinella* | 2.2% | 0.01 | 3.22 | 5 | 262 | 40.1% | 0.001 |
| Caulobacterales | Hyphomonadaceae | unknown genus | 2.0% | 0.01 | 3.38 | 12 | 253 | 42.7% | 0.001 |
| Chitinophagales | unknown family | unknown genus | 1.7% | 0.01 | 2.03 | 2 | 205 | 44.9% | 0.011 |
| Rhodobacterales | Rhodobacteraceae | unknown genus | 1.7% | 0.01 | 2.12 | 100 | 302 | 47.0% | 0.622 |
| Rhodobacterales | Rhodobacteraceae | *Planktotalea* | 1.6% | 0.01 | 2.72 | 244 | 50 | 49.1% | 0.003 |

## Table S6. SIMPER results of the most contributing genera to the dissimilarities between water samples and algal samples collected at Tamaris. Only the first 50% of the cumulative contribution was showed. *p* values were calculated with a permutation test constructed with 999 permutations.

| **Order** | **Family** | **Genus** | **Average contribution** | **SD** | **Ratio** | **Av. water samples** | **Av. thallus samples** | **Cum. sum** | ***p* value** |
| --- | --- | --- | --- | --- | --- | --- | --- | --- | --- |
| Synechococcales | Cyanobiaceae | *Synechococcus* | 9.0% | 0.07 | 1.24 | 1067 | 1 | 10.3% | 0.063 |
| SAR11 clade | Clade I | Clade Ia | 3.9% | 0.05 | 0.81 | 462 | 0 | 14.7% | 0.003 |
| Thiohalorhabdales | Thiohalorhabdaceae | *Granulosicoccus* | 3.3% | 0.02 | 1.72 | 13 | 407 | 18.5% | 0.019 |
| Chitinophagales | Saprospiraceae | unknown genus | 3.3% | 0.02 | 2.18 | 16 | 406 | 22.3% | 0.025 |
| Flavobacteriales | Flavobacteriaceae | NS5 marine group | 3.2% | 0.02 | 1.75 | 385 | 1 | 26.0% | 0.002 |
| Rhodobacterales | Rhodobacteraceae | unknown genus | 3.2% | 0.02 | 1.40 | 80 | 447 | 29.6% | 0.027 |
| Caulobacterales | Hyphomonadaceae | *Litorimonas* | 2.8% | 0.03 | 0.94 | 2 | 331 | 32.8% | 0.125 |
| Pirellulales | Pirellulaceae | unknown genus | 2.8% | 0.03 | 1.02 | 329 | 0 | 36.0% | 0.017 |
| Synechococcales | Synechococcales Incertae Sedis | *Schizothrix* | 2.7% | 0.03 | 0.78 | 3 | 318 | 39.0% | 0.141 |
| Flavobacteriales | Flavobacteriaceae | NS4 marine group | 2.2% | 0.01 | 1.53 | 266 | 1 | 41.5% | 0.002 |
| Rhizobiales | Rhizobiaceae | *Nitratireductor* | 1.9% | 0.04 | 0.52 | 22 | 232 | 43.7% | 0.144 |
| Rhodobacterales | Rhodobacteraceae | *Planktomarina* | 1.7% | 0.01 | 2.32 | 202 | 1 | 45.7% | 0.001 |
| Rhodobacterales | Rhodobacteraceae | HIMB11 | 1.6% | 0.02 | 0.72 | 191 | 0 | 47.5% | 0.006 |
| Betaproteobacteriales | Burkholderiaceae | *Limnobacter* | 1.6% | 0.02 | 0.69 | 191 | 0 | 49.3% | 0.096 |
| Cellvibrionales | Halieaceae | OM60(NOR5) clade | 1.4% | 0.01 | 1.60 | 171 | 2 | 51.0% | 0.002 |

## Table S7. SIMPER results of the most contributing genera to the dissimilarities between rocky biofilms and algal samples collected at Carqueiranne. Only the first 50% of the cumulative contribution was showed. *p* values were calculated with a permutation test constructed with 999 permutations.

| **Order** | **Family** | **Genus** | **Average contribution** | **SD** | **Ratio** | **Av. rocky biofilms** | **Av. thallus samples** | **Cum. sum** | ***p* value** |
| --- | --- | --- | --- | --- | --- | --- | --- | --- | --- |
| Flavobacteriales | Flavobacteriaceae | *Algitalea* | 5.1% | 0.03 | 1.86 | 2 | 609 | 8.8% | 0.005 |
| Rhodobacterales | Rhodobacteraceae | *Loktanella* | 2.3% | 0.02 | 1.48 | 334 | 264 | 12.7% | 0.074 |
| Chitinophagales | Saprospiraceae | unknown genus | 2.2% | 0.01 | 1.60 | 479 | 435 | 16.4% | 0.441 |
| unknown order | unknown family | unknown genus | 1.7% | 0.02 | 0.88 | 216 | 46 | 19.4% | 0.023 |
| Thalassobaculales | unknown family | unknown genus | 1.6% | 0.01 | 1.35 | 195 | 0 | 22.2% | 0.014 |
| Flavobacteriales | Flavobacteriaceae | *Aquimarina* | 1.6% | 0.02 | 0.83 | 196 | 57 | 24.9% | 0.129 |
| Chitinophagales | unknown family | unknown genus | 1.5% | 0.01 | 1.79 | 25 | 205 | 27.5% | 0.036 |
| Caulobacterales | Hyphomonadaceae | unknown genus | 1.4% | 0.01 | 2.14 | 81 | 253 | 30.0% | 0.053 |
| Flavobacteriales | Flavobacteriaceae | *Croceitalea* | 1.4% | 0.01 | 1.78 | 30 | 197 | 32.4% | 0.05 |
| Verrucomicrobiales | Rubritaleaceae | *Rubritalea* | 1.3% | 0.01 | 1.16 | 4 | 155 | 34.6% | 0.33 |
| Flavobacteriales | Flavobacteriaceae | *Maritimimonas* | 1.1% | 0.01 | 1.46 | 140 | 5 | 36.5% | 0.002 |
| Chitinophagales | Saprospiraceae | *Lewinella* | 1.0% | 0.01 | 1.54 | 225 | 262 | 38.2% | 0.758 |
| Rhodobacterales | Rhodobacteraceae | unknown genus | 0.9% | 0.01 | 1.25 | 248 | 302 | 39.8% | 0.996 |
| Nostocales | Xenococcaceae | unknown genus | 0.8% | 0.01 | 0.70 | 97 | 0 | 41.2% | 0.034 |
| Nostocales | Xenococcaceae | *Chroococcidiopsis* | 0.8% | 0.01 | 0.72 | 97 | 2 | 42.6% | 0.078 |
| Nitrosopumilales | Nitrosopumilaceae | *Candidatus* *Nitrosopumilus* | 0.8% | 0.01 | 0.70 | 90 | 0 | 43.9% | 0.07 |
| Steroidobacterales | Woeseiaceae | JTB255 marine benthic group | 0.7% | 0.01 | 0.70 | 88 | 0 | 45.2% | 0.034 |
| Sphingomonadales | Sphingomonadaceae | *Erythrobacter* | 0.7% | 0.00 | 1.67 | 109 | 63 | 46.4% | 0.024 |
| Flavobacteriales | Flavobacteriaceae | *Tenacibaculum* | 0.7% | 0.00 | 2.49 | 1 | 83 | 47.6% | 0.297 |
| Steroidobacterales | Woeseiaceae | *Woeseia* | 0.6% | 0.01 | 1.00 | 80 | 8 | 48.7% | 0.007 |
| Pirellulales | Pirellulaceae | *Rhodopirellula* | 0.6% | 0.00 | 2.15 | 89 | 14 | 49.8% | 0.017 |

## Table S8. SIMPER results of the most contributing genera to the dissimilarities between rocky biofilms and algal samples collected at Tamaris. Only the first 50% of the cumulative contribution is showed. *p* values were calculated with a permutation test constructed with 999 permutations.

| **Order** | **Family** | **Genus** | **Average contribution** | **sd** | **ratio** | **Av. rocky biofilms** | **Av. thallus samples** | **Cum. sum** | ***p* value** |
| --- | --- | --- | --- | --- | --- | --- | --- | --- | --- |
| Thiohalorhabdales | Thiohalorhabdaceae | *Granulosicoccus* | 2.8% | 0.02 | 1.40 | 79 | 407 | 4.2% | 0.045 |
| Synechococcales | Synechococcales Incertae Sedis | *Schizothrix* | 2.5% | 0.03 | 0.74 | 16 | 318 | 8.0% | 0.143 |
| Caulobacterales | Hyphomonadaceae | *Litorimonas* | 2.4% | 0.03 | 0.84 | 85 | 331 | 11.6% | 0.155 |
| Chitinophagales | Saprospiraceae | unknown genus | 2.1% | 0.01 | 1.52 | 172 | 406 | 14.8% | 0.448 |
| Rhodobacterales | Rhodobacteraceae | unknown genus | 2.1% | 0.02 | 1.38 | 338 | 447 | 18.0% | 0.296 |
| Flavobacteriales | Flavobacteriaceae | *Aquimarina* | 2.1% | 0.01 | 1.41 | 279 | 36 | 21.2% | 0.022 |
| Rhizobiales | Rhizobiaceae | *Nitratireductor* | 1.9% | 0.04 | 0.51 | 7 | 232 | 24.1% | 0.148 |
| Rhodobacterales | Rhodobacteraceae | *Loktanella* | 1.8% | 0.01 | 1.34 | 278 | 103 | 26.8% | 0.264 |
| Nostocales | Xenococcaceae | *Pleurocapsa* | 1.8% | 0.02 | 0.95 | 226 | 67 | 29.5% | 0.018 |
| unknown order * | unknown family | unknown genus | 1.5% | 0.01 | 1.08 | 208 | 77 | 31.7% | 0.024 |
| Thalassobaculales | unknown family | unknown genus | 1.4% | 0.02 | 0.73 | 167 | 0 | 33.8% | 0.074 |
| Rhodobacterales | Rhodobacteraceae | *Sulfitobacter* | 1.4% | 0.01 | 1.69 | 188 | 23 | 35.9% | 0.008 |
| Flavobacteriales | Flavobacteriaceae | *Algitalea* | 1.3% | 0.01 | 1.26 | 184 | 144 | 37.9% | 0.975 |
| Verrucomicrobiales | Rubritaleaceae | *Rubritalea* | 1.3% | 0.02 | 0.86 | 12 | 166 | 39.9% | 0.33 |
| Flavobacteriales | Flavobacteriaceae | *Winogradskyella* | 1.1% | 0.01 | 0.90 | 116 | 99 | 41.6% | 0.162 |
| Verrucomicrobiales | Rubritaleaceae | *Roseibacillus* | 1.1% | 0.01 | 1.02 | 69 | 134 | 43.2% | 0.125 |
| Cytophagales | Amoebophilaceae | *Candidatus* *Amoebophilus* | 1.0% | 0.01 | 0.90 | 115 | 0 | 44.7% | 0.002 |
| Pirellulales | Pirellulaceae | *Blastopirellula* | 1.0% | 0.01 | 1.38 | 103 | 156 | 46.1% | 0.329 |
| Flavobacteriales | Flavobacteriaceae | *Tenacibaculum* | 0.9% | 0.01 | 1.75 | 76 | 105 | 47.5% | 0.028 |
| Sphingomonadales | Sphingomonadaceae | *Sphingorhabdus* | 0.9% | 0.00 | 1.82 | 28 | 131 | 48.8% | 0.024 |
| Phormidesmiales | Phormidesmiaceae | *Acrophormium* | 0.9% | 0.01 | 1.15 | 7 | 110 | 50.2% | 0.13 |

*unknown Bacteria

## Table S9. SIMPER results of the most contributing genera to the dissimilarities between basal and apical parts of algal samples collected at Carqueiranne. Only the first 50% of the cumulative contribution is showed. *p* values were calculated with a permutation test constructed with 999 permutations.

| **Order** | **Family** | **Genus** | **Average contribution** | **SD** | **Ratio** | **Av. basal parts** | **Av. apical parts** | **Cum. sum** | ***p* value** |
| --- | --- | --- | --- | --- | --- | --- | --- | --- | --- |
| Flavobacteriales | Flavobacteriaceae | *Algitalea* | 4.1% | 0.01 | 3.17 | 417 | 609 | 9.6% | 0.118 |
| Rhodobacterales | Rhodobacteraceae | *Loktanella* | 2.3% | 0.01 | 2.67 | 142 | 410 | 14.9% | 0.166 |
| Verrucomicrobiales | Rubritaleaceae | *Rubritalea* | 1.9% | 0.01 | 1.39 | 44 | 265 | 19.2% | 0.185 |
| Flavobacteriales | Flavobacteriaceae | *Croceitalea* | 1.6% | 0.00 | 4.49 | 106 | 301 | 23.1% | 0.033 |
| Chitinophagales | unknown family | unknown genus | 1.6% | 0.01 | 2.20 | 296 | 108 | 26.8% | 0.062 |
| Rhodobacterales | Rhodobacteraceae | unknown genus | 1.4% | 0.01 | 2.43 | 391 | 227 | 30.0% | 0.742 |
| Caulobacterales | Hyphomonadaceae | unknown genus | 1.1% | 0.00 | 3.23 | 344 | 214 | 32.6% | 0.501 |
| Chitinophagales | Saprospiraceae | unknown genus | 1.1% | 0.01 | 1.54 | 467 | 346 | 35.1% | 0.965 |
| Flavobacteriales | Flavobacteriaceae | unknown genus | 1.0% | 0.01 | 2.01 | 61 | 181 | 37.5% | 0.023 |
| Rhodovibrionales | Kiloniellaceae | unknown genus | 0.8% | 0.00 | 2.84 | 9 | 105 | 39.4% | 0.002 |
| Flavobacteriales | Flavobacteriaceae | *Aquimarina* | 0.8% | 0.01 | 0.97 | 20 | 109 | 41.2% | 0.51 |
| unknown order | unknown family | unknown genus | 0.7% | 0.00 | 2.40 | 94 | 11 | 42.8% | 0.179 |
| Rhodobacterales | Rhodobacteraceae | *Octadecabacter* | 0.6% | 0.00 | 7.13 | 33 | 110 | 44.3% | 0.018 |
| Flavobacteriales | Flavobacteriaceae | *Winogradskyella* | 0.6% | 0.01 | 0.89 | 77 | 142 | 45.8% | 0.385 |
| Phormidesmiales | Phormidesmiaceae | *Acrophormium* | 0.6% | 0.00 | 1.79 | 88 | 16 | 47.2% | 0.398 |
| Thiohalorhabdales | Thiohalorhabdaceae | *Granulosicoccus* | 0.5% | 0.00 | 3.91 | 96 | 32 | 48.5% | 0.829 |
| Verrucomicrobiales | DEV007 | unknown genus | 0.5% | 0.00 | 1.98 | 82 | 19 | 49.7% | 0.032 |

## Table S10. SIMPER results of the most contributing genera to the dissimilarities between basal and apical parts of algal samples collected at Tamaris. Only the first 50% of the cumulative contribution is showed. *p* values were calculated with a permutation test constructed with 999 permutations.

| **Order** | **Family** | **Genus** | **Average contribution** | **sd** | **ratio** | **Av. basal part** | **Av. apical part** | **Cum. sum** | ***p* value** |
| --- | --- | --- | --- | --- | --- | --- | --- | --- | --- |
| Rhizobiales | Rhizobiaceae | *Nitratireductor* | 5.3% | 0.05 | 1.05 | 5 | 638 | 8.8% | 0.002 |
| Caulobacterales | Hyphomonadaceae | *Litorimonas* | 5.3% | 0.03 | 1.78 | 92 | 721 | 17.6% | 0.002 |
| Synechococcales | Synechococcales Incertae Sedis | *Schizothrix* | 3.8% | 0.05 | 0.77 | 69 | 512 | 23.9% | 0.129 |
| Rhodobacterales | Rhodobacteraceae | unknown genus | 2.5% | 0.02 | 1.60 | 482 | 213 | 28.1% | 0.22 |
| Thiohalorhabdales | Thiohalorhabdaceae | *Granulosicoccus* | 2.4% | 0.02 | 1.44 | 403 | 467 | 32.1% | 0.257 |
| Flavobacteriales | Flavobacteriaceae | *Winogradskyella* | 1.9% | 0.02 | 0.84 | 56 | 217 | 35.2% | 0.131 |
| Chitinophagales | Saprospiraceae | unknown genus | 1.8% | 0.01 | 1.61 | 426 | 402 | 38.2% | 0.704 |
| Verrucomicrobiales | Rubritaleaceae | *Roseibacillus* | 1.8% | 0.01 | 1.22 | 30 | 242 | 41.1% | 0.017 |
| Flavobacteriales | Flavobacteriaceae | *Algitalea* | 1.5% | 0.01 | 1.38 | 222 | 41 | 43.6% | 0.815 |
| Chitinophagales | Saprospiraceae | *Portibacter* | 1.5% | 0.01 | 2.04 | 236 | 59 | 46.1% | 0.017 |
| Nostocales | Xenococcaceae | *Pleurocapsa* | 1.2% | 0.01 | 1.41 | 157 | 18 | 48.0% | 0.232 |
| Chitinophagales | Saprospiraceae | *Lewinella* | 1.1% | 0.00 | 4.24 | 174 | 39 | 49.9% | 0.522 |

## Table S11. Summary of PERMANOVA results examining the effect of “site” and “thallus parts” factors for each normalized metabolomics dataset.

D. f ; MS ; F ; R^2^ and P correspond to degrees of freedom ; mean square ; F ratio ; coefficient of determination and *p* value, respectively

|  | | **Surface extracts** | | | | | **Total extracts** | | | | |
| --- | --- | --- | --- | --- | --- | --- | --- | --- | --- | --- | --- |
|  |  | **D. f** | **MS** | **F** | **R^2^** | **P** | **D. f** | **MS** | **F** | **R^2^** | **P** |
| **LC-(+)-ESI-MS** | Site | 1 | 4690 | 2.383 | 0.08725 | 0.002 | 1 | 14505 | 8.209 | 0.236 | 0.001 |
|  | Thallus parts | 2 | 3997 | 2.031 | 0.14869 | 0.001 | 2 | 4747 | 2.687 | 0.154 | 0.002 |
|  | Site:Thallus parts | 2 | 2825 | 1.436 | 0.10511 | 0.017 | 2 | 2828 | 1.601 | 0.092 | 0.036 |
|  | Residuals | 18 | 1968 |  | 0.65895 |  | 18 | 1767 |  | 0.517 |  |
|  | Total | 23 |  |  | 1 |  | 23 |  |  | 1 |  |
| **LC-(-)-ESI-MS** | Site | 1 | 367 | 5.371 | 0.1426 | 0.001 | 1 | 1890 | 28.2 | 0.471 | 0.001 |
|  | Thallus parts | 2 | 350.1 | 5.124 | 0.27204 | 0.001 | 2 | 300.1 | 4.48 | 0.15 | 0.003 |
|  | Site:Thallus parts | 2 | 138.3 | 2.025 | 0.1075 | 0.026 | 2 | 156.5 | 2.335 | 0.078 | 0.049 |
|  | Residuals | 18 | 68.33 | 0.478 |  |  | 18 | 67 | 0.301 |  |  |
|  | Total | 23 |  |  | 1 |  | 23 |  |  | 1 |  |
| **GC-MS** | Site | 1 | 16926 | 1.171 | 0.04856 | 0.248 | 1 | 2669 | 5.757 | 0.18 | 0.002 |
|  | Thallus parts | 2 | 28282 | 1.957 | 0.1623 | 0.009 | 2 | 1550 | 3.344 | 0.209 | 0.002 |
|  | Site:Thallus parts | 2 | 14675 | 1.015 | 0.08421 | 0.4 | 2 | 582.3 | 1.256 | 0.079 | 0.243 |
|  | Residuals | 17 | 14452 |  | 0.70493 |  | 17 | 463.6 |  | 0.532 |  |
|  | Total | 22 |  |  | 1 |  | 22 |  |  | 1 |  |

## Table S12. Multivariate pairwise results (*p* values) examining differences between both sites and between each thallus parts for each metabolomics dataset.

|  |  | **Surface extracts** | | | **Total extracts** | | |
| --- | --- | --- | --- | --- | --- | --- | --- |
| **Comparison type** | Samples compared | LC-(+)-ESI-MS | LC-(-)-ESI-MS | GC-MS | LC-(+)-ESI-MS | LC-(-)-ESI-MS | GC-MS |
| **Site comparison** | Carqueiranne vs Tamaris | 0.001 | 0.005 | 0.258 | 0.001 | 0.001 | 0.002 |
| **Sample type comparison** | Basal vs median parts | 0.006 | 0.013 | 0.294 | 0.0345 | 0.161 | 0.0135 |
|  | Basal vs Apical parts | 0.003 | 0.003 | 0.003 | 0.033 | 0.161 | 0.0135 |
|  | Median vs apical parts | 0.058 | 0.013 | 0.0375 | 0.145 | 0.161 | 0.278 |

## Table S13. Summary of R^2^ values obtained for the two first components after cross validation test for PLS-DA obtained with each metabolomics dataset.

The method “leave-one-out cross-validation” (LOOCV) was chosen for the cross-validation.

|  | **Surface extracts** | **Total extracts** |
| --- | --- | --- |
| **LC-(+)-ESI-MS** | 0.972 | 0.970 |
| **LC-(-)-ESI-MS** | 0.866 | 0.907 |
| **GC-MS** | 0.717 | 0.858 |

## Table S14. List of compounds identified through GC-MS analyses

| **Name** | **Molecular formula** | **Retention time (s)** | **Experimental RI** | **RI from reference** | **Reference** | **Match with NIST11 database (%)** |
| --- | --- | --- | --- | --- | --- | --- |
| *α*-cubebene | C_15_H_24_ | 23.639 | 1351 | 1352 | Lucero, Fredrickson, et al., 2006 | 99 |
| *β*-bourbonene | C_15_H_24_ | 24.584 | 1386 | 1387 | Adams, 2008 | 97 |
| *β*-cubebene | C_15_H_24_ | 24.717 | 1391 | 1387 | Adams, 2008 | 89 |
| *β*-ylangene | C_15_H_24_ | 25.495 | 1421 | 1421 | Flamini, Luigi Cioni, et al., 2005 | 99 |
| *β*-copaene | C_15_H_24_ | 25.723 | 1429 | 1430 | Adams, 2008 | 97 |
| *trans*-muurola-3,5-diene | C_15_H_24_ | 26.252 | 1452 | 1451 | Adams, 2008 | 90 |
| *cis*-muurola-4(14),5-diene | C_15_H_24_ | 26.673 | 1469 | 1465 | Adams, 2008 | 72 |
| *trans*-cadina-1(6),4-diene | C_15_H_24_ | 26.84 | 1476 | 1475 | Adams, 2008 | 93 |
| *γ*-muurolene | C_15_H_24_ | 26.929 | 1479 | 1477 | Flamini, Cioni, et al., 2007 | 99 |
| *α*-amorphene & germacrene D | C_15_H_24_ | 26.994 | 1482 | 1483-1484 | Adams, 2008 | 93 |
| *α*-muurolene | C_15_H_24_ | 27.489 | 1502 | 1501 | Flamini, Tebano, et al., 2006 | 99 |
| *cis*-calamenene | C_15_H_22_ | 28.026 | 1525 | 1522 | Palmeira, Moura, et al., 2004 | 96 |
| *trans*-cadina-1,4-diene | C_15_H_24_ | 28.295 | 1536 | 1536 | Andrade, Sampaio, et al., 2007 | 93 |
| *α*-calacorene | C_15_H_20_ | 28.45 | 1543 | 1543 | Lucero, Estell, et al., 2003 | 53 |
| unidentified sesquiterpene | C_15_H_22_ | 28.547 | 1547 | - | - | - |
| unidentified sesquiterpene | C_15_H_24_O | 28.895 | 1561 | - | - | - |
| *β*-calacorene | C_15_H_20_ | 29.004 | 1566 | 1564 | Javidnia, Miri, et al., 2006 | 96 |
| unidentified sesquiterpene | C_15_H_24_O_2_ | 29.103 | 1570 | - | - | - |
| gleenol isomer (?) | C_15_H_26_O | 29.261 | 1577 | - | - | - |
| gleenol | C_15_H_26_O | 29.619 | 1587 | 1587 | Custer, 2009 | 94 |
| unidentified sesquiterpene | C_15_H_24_O_2_ | 33.139 | 1749 | - | - | - |
| unidentified sesquiterpene | C_15_H_24_O_2_ | 33.258 | 1755 | - | - | - |
| unidentified sesquiterpene | C_15_H_24_O_2_ | 33.354 | 1760 | - | - | - |
| unidentified sesquiterpene | C_15_H_24_O_2_ | 34.322 | 1808 | - | - | - |

## Table S15. Putative annotation of VIPs from the LC-(+)-ESI-MS dataset of samples of *T. atomaria* collected at Tamaris

| **VIP N°** | ***m/z*** | **RT (s)** | **VIP score** | **Molecular formula** | **Mass error (ppm)** | **mσ^a^** | **MS/MS fragment ions (relative abundance in %)** | **Putative identification^b^** |
| --- | --- | --- | --- | --- | --- | --- | --- | --- |
| 1 | 450.3355 | 684 | 3.8 | C_30_H_44_NO_2_ | 1.1 | 5.5 | 450.3355 [M+NH_4_]^+^ (2), 201.1641 [C_15_H_21_]^+^ (69), 159.1166 [C_12_H_15_]^+^ (55), 145.1011 [C_11_H_13_]^+^ (100), 119.0854 [C_9_H_11_]^+^ (27), 95.0856 [C_7_H_11_]^+^ (30) | C_30_H_40_O_2_ (*apo*-carotenoid?) |
| 2 | 318.2790 | 587 | 3.7 | C_21_H_36_NO | 0.2 | 3.0 | 318.2790 [M+H]^+^ (3), 286.2514 [C_20_H_32_N]^+^ (2), 175.1458 [C_13_H_19_]^+^ (4), 149.1321 [C_11_H_17_]^+^ (5), 126.0909 [C_7_H_12_NO]^+^ (36), 108.0802 [C_7_H_10_N]^+^ (18), 95.0860 [C_7_H_11_]^+^ (23), 93.0695 [C_7_H_9_]^+^ (19), 81.0699 [C_6_H_9_]^+^ (100), 69.0697 [C_5_H_9_]^+^ (52) | 3,4-epoxy-lobophorene B? |
| 3 | 682.5617 | 683 | 3.0 | C_40_H_76_NO_7_ | -0.4 | 5.5 | n.o. | DGTA (C30:1) |
| 4 | 482.3475 | 584 | 2.9 | C_27_H_48_NO_6_ | 0.2 | 2.5 | 482.3475 [M+NH_4_]^+^ (1), 271.2415 [C_20_H_31_]^+^ (90), 215.1791 [C_16_H_23_]^+^ (87), 201.1635 [C_15_H_21_]^+^ (69), 189.1638 [C_14_H_21_]^+^ (45), 175.1481 [C_13_H_19_]^+^ (73), 161.1322 [C_12_H_17_]^+^ (93), 135.1166 [C_10_H_15_]^+^ (77), 109.1011 [C_8_H_13_]^+^ (100), 95.0857 [C_7_H_11_]^+^ (69), 81.0698 [C_6_H_9_]^+^ (81), 69.0695 [C_5_H_9_]^+^ (57) | GGG derivative |
| 5 | 474.3793 | 546 | 2.7 | C_26_H_52_NO_6_ | 0.1 | 11.0 | 474.3793 [M+H]^+^ (100), 456.3688 [M-H_2_O+H]^+^ (22), 412.3775 [C_25_H_50_NO_3_]^+^ (1), 369.3021 [C_22_H_41_O_4_]^+^ (1), 313.2733 [C_19_H_37_O_3_]^+^ (1), 236.1491 [C_10_H_22_NO_5_]^+^ (57) | *lyso*-DGTA (C16:0) |
| 6 | 421.3167 | 573 | 2.6 | C_22_H_45_O_7_ | -1.0 | 11.0 | 421.3167 [M+H]^+^ (1), 385.2950 [M-2H_2_O+H]^+^ (2), 239.2372 [C_16_H_31_O]^+^ (4), 165.0759 [C_6_H_13_O_5_]^+^ (14), 147.0653 [C_6_H_11_O_4_]^+^ (18), 129.0547 [C_6_H_9_O_3_]^+^ (35), 111.0445 [C_6_H_7_O_2_]^+^ (20), 99.0445 [C_5_H_7_O_2_]^+^ (21), 95.0858 [C_7_H_11_]^+^ (20), 83.0494 [C_5_H_7_O]^+^ (34), 69.0338 [C_4_H_5_O]^+^ (100), 57.0704 [C_4_H_9_]^+^ (26) | Mannitol + FA chain (C16 :0)? |
| 7 | 623.2865 | 653 | 2.5 | C_35_H_43_O_10_ | -1.0 | 8.5 | 623.2865 [M+H]^+^ (100), 605.2755 [M-H_2_O+H]^+^ (67), 591.2604 [C_34_H_39_O_9_]^+^ (4), 573.2496 [C_34_H_37_O_8_]^+^ (12), 545.2546 [C_33_H_37_O_7_]^+^ (48), 217.1948 [C_16_H_25_]^+^ (9) | C_35_H_42_O_10_ |
| 8 | 524.3941 | 503 | 2.2 | C_30_H_54_NO_6_ | -0.3 | 2.2 | 524.3941 [M+H]^+^ (100), 363.2886 [C_23_H_39_O_3_]^+^ (0,5), 236.1496 [C_10_H_22_NO_5_]^+^ (5) | *lyso-*DGTA (C20:3) |
| 9 | 423.3106 | 562 | 2.2 | C_25_H_43_O_5_ | 0.4 | 26.8 | n.o. | C_25_H_42_O_5_ |
| 10 | 375.2167 | 458 | 2.1 | C_22_H_31_O_5_ | -0.1 | 16.8 | 375.2167 [M+H]^+^ (1), 315.1928 [C_20_H_27_O_3_]^+^ (1), 205.0858 [C_12_H_13_O_3_]^+^ (7), 177.0909 [C_11_H_13_O_2_]^+^ (12), 159.0804 [C_11_H_11_O]^+^ (23), 131.0853 [C_10_H_11_]^+^ (14), 109.1012 [C_8_H_13_]^+^ (100), 95.0493 [C_6_H_7_O]^+^ (8), 67.0545 [C_5_H_7_]^+^ (7) | C_22_H_30_O_5_ (Acetylated diterpene) |
| 11 | 135.0471 | 50 | 2.01 | C_5_H_11_O_2_S | 2.0 | 6.9 | 135.0471 [M+H]^+^ (19), 73.0283 [C_3_H_5_O_2_]^+^ (100), 63.0261 [C_2_H_7_S]^+^ (100), 61.0108 [C_2_H_5_S]^+^ (5), 55.0177 [C_3_H_3_O]^+^ (7) | DMSP^d^ |
| 12 | 307.1517 | 378 | 1.9 | C_17_H_23_O_5_ | 7.2 | 18.4 | 307.1517 [M+H]^+^ (100), 215.1039 [C_14_H_15_O_2_]^+^ (3), 121.1021 [C_9_H_13_]^+^ (6), 115.0363 [C_5_H_7_O_3_]^+^ (8), | C_17_H_22_O_5_ |
| 13 | 808.6099 | 628 | 1.9 | C_47_H_87_NO_7_P | 14.4 | 7.9 | 808.6099 [M+H]^+^ (100), 522.3794 [C_27_H_57_NO_6_P]^+^ (2), 504.3681 [C_27_H_55_NO_5_P]^+^ (2), 184.07 [C_5_H_15_NO_4_P]^+^ (13) | PC (C39:5)? |
| 14 | 741.6138 | 618 | 1.9 | C_44_H_86_O_6_P | 2.7 | 11.1 | 741.6138 [M+H]+ (100), 697.6233 [C_43_H_86_O_4_P]+ (1), 571.4716 [C_34_H_68_O_4_P]+ (1), 439.3891 [C_24_H_56_O_4_P]^+^ (9), | C_44_H_85_O_6_P |
| 15 | 577.5193 | 732 | 1.9 | C_37_H_69_O_4_ | -0.4 | 21.1 | 577.5193 [M+H]^+^ (100), 265.2523 [C_18_H_33_O]^+^ (23), 247.2414 [C_18_H_31_]^+^ (16), 239.2358 [C_16_H_31_O]^+^ (25), 221.2275 [C_16_H_29_]^+^ (3), 149.1329 [C11H17]^+^ (9] , 135.1162 [C_10_H_15_]^+^ (10) , 123.1158 [C_9_H_15_]^+^ (17) , 109.1011 [C_8_H_13_]^+^ (32), 95.0858 [C_7_H_11_]^+^ (48), 83.0857 [C_6_H_11_]^+^ (32) , 69.0699 [C_5_H_9_]^+^ (28), 57.0698 [C_4_H_9_]^+^ (14) | DG (C34:1) (C18:1, C16:0) |
| 16 | 704.5474 | 661 | 1.9 | C_42_H_74_NO_7_ | 0.8 | 8.2 | 704.5474 [M+H]^+^ (100), 494.3778 [C_28_H_48_NO_6_]^+^ (16), 446.3480 [C_24_H_48_NO_6_]^+^ (42), 236.1496 [C_10_H_22_NO_5_]^+^ (5) | DGTA (C32:0) (C18:4, C14:0) |

^a^ Constructor statistical match factor (comparison of theoretical and experimental isotopic patterns); ^b^ Abbreviations: PC: phosphatidylcholine, DGTA: diacylglycerylhydroxymethyl-*N*,*N*,*N*-trimethyl-*β*-alanine, DG: diacylglycerol, GGG: geranylgeranylglycerol, FA: Fatty Acid; ^c^ not observed; ^d^ This identification was confirmed with a commercial standard.

## Table S16. Putative annotation of VIPs from the LC-(+)-ESI-MS dataset of samples of *T. atomaria* collected at Carqueiranne

| **VIP N°** | ***m/z*** | **RT (s)** | **VIP score** | **Formula** | **Mass error (ppm)** | **mσ^a^** | **MS/MS fragment ions (relative abundance in %)** | **Putative identification^b^** |
| --- | --- | --- | --- | --- | --- | --- | --- | --- |
| 1 | 704.5468 | 607 | 3.1 | C_39_H_79_NO_7_P | 17.1 | 17 | 604.5468 [M+H]^+^ (100), 494.3487 [C_25_H_53_NO_6_P]^+^ (1), 476.3371 [C_25_H_51_NO_5_P]^+^ (1), 184.0722 [C_5_H_15_NO_4_P]^+^ (1) | PC (C31:1)? |
| 2 | 706.5629 | 673 | 3.1 | C_42_H_76_NO_7_ | -1.4 | 10.2 | 706.5629 [M+H]^+^ (100), 496.3637 [C_28_H_50_NO_6_]^+^ (15), 494.3480 [C_28_H_48_NO_6_]^+^ (1), 478.3536 [C_28_H_48_NO_5_]^+^ (4), 446.3482 [C_24_H_48_NO_6_]^+^ (40), 428.3371 [C_24_H_46_NO_5_]^+^ (4), 236.1494 [C_10_H_22_NO_5_]^+^ (4) | DGTA (C32:3) (C14:0, C18:3) |
| 3 | 758.5936 | 630 | 3.0 | C_43_H_85_NO_7_P | 16.3 | 10.2 | 758.5936 [M+H]^+^ (100), 520.3619 [C_27_H_55_NO_6_P]^+^ (1), 502.3523 [C_27_H_53_NO_5_P]^+^ (1), 184.0733 [C_5_H_15_NO_4_P]^+^ (8), | PC (C35:2)? |
| 4 | 405.3519 | 694 | 2.9 | C_30_H_45_ | -3.7 | 21.5 | 405.3519 [M+H]^+^ (100), 321.2574 [C_24_H_33_]^+^ (5), 201.1641 [C_15_H_21_]^+^ (100), 145.1009 [C_11_H_13_]^+^ (6), 135.1168 [C_10_H_15_]^+^ (17), 95.0857 [C_7_H_11_]^+^ (7), 93.0702 [C_7_H_9_]^+^ (8) | C_30_H_44_ (*apo*-carotenoid?) |
| 5 | 810.6252 | 636 | 2.9 | C_47_H_89_NO_7_P | 15.3 | 5.1 | 810.6252 [M+H]^+^ (100), 522.3797 [C_27_H_57_NO_6_P]^+^ (2), 504.3683 [C_27_H_55_NO_5_P]^+^ (2), 184.0737 [C_5_H_15_NO_4_P]^+^ (40) | PC (C39:4)? |
| 6 | 778.5619 | 602 | 2.8 | C_45_H_81_NO_7_P | 14.6 | 8.7 | 778.5619 [M+H]^+^ (100), 520.3585 [C_27_H_55_NO_6_P]^+^ (1), 502.3549 [C_27_H_53_NO_5_P]^+^ (1), 184.0732 [C_5_H_15_NO_4_P]^+^ (17) | PC (C37:6)? |
| 7 | 542.3247 | 505 | 2.6 | C_28_H_49_NO_7_P | -1 | 8.8 | 542.3247 [M+H]^+^ (100), 524.3157 [M-H_2_O+H]^+^ (1), 184.0732 [C_5_H_15_NO_4_P]^+^ (100) | *lyso*-PC (C20:5) |
| 8 | 732.5781 | 623 | 2.5 | C_41_H_83_NO_7_P | 16.2 | 6.3 | 732.5781 [M+H]^+^ (100), 522.3788 [C_27_H_57_NO_6_P]^+^ (1), 504.3694 [C_27_H_55_NO_5_P]^+^ (1), 446.3471 [C_21_H_53_NO_6_P]^+^ (1), 428.3370 [C_21_H_51_NO_5_P]^+^ (1), 184.0735 [C_5_H_15_NO_4_P]^+^ (1) | PC (C33:1)? |
| 9 | 496,3637 | 486 | 2.4 | C_28_H_50_NO_6_ | -0.2 | 8.6 | 496.3637 [M+H]^+^ (100), 478.3534 [C_28_H_48_NO_5_]^+^ (1), 391.2830 [C_24_H_39_O_4_]^+^ (1), 335.2590 [C_21_H_35_O_3_]^+^ (1), 236.1494 [C_10_H_22_NO_5_]^+^ (4), 218.1386 [C_10_H_20_NO_4_]^+^ (1), 144.1020 [C_7_H_14_NO_2_]^+^ (1), 100.1126 [C_6_H_14_N]^+^ (2) | *lyso*-DGTA (C18:3) |
| 10 | 203.1793 | 531 | 2.4 | C_15_H_23_ | 0.7 | 17.4 | 203.1793 [M+H]^+^ (31), 161.1321 [C_12_H_17_]^+^ (26), 147.1167 [C_11_H_15_]^+^ (71), 133.1010 [C_10_H_13_]^+^ (32), 119.0855 [C_9_H_11_]^+^ (50), 109.1011 [C_8_H_13_]^+^ (27), 105.0698 [C_8_H_9_]^+^ (83), 91.0544 [C_7_H_7_]^+^ (46), 81.0697 [C_6_H_9_]^+^ (81), 69.0697 [C_5_H_9_]^+^ (56), 55.0541 [C_4_H_7_]^+^ (14) | Germacra-4(15),5,10(14)-trien-9-ol  [M-H_2_O+H]^+ d^ |
| 11 | 376.2598 | 486 | 2.2 | C_26_H_34_NO | 9.5 | 7.7 | n.o.^c^ | C_26_H_33_NO? |
| 12 | 406.3598 | 750 | 2.2 | C_29_H_44_N | -33.4 | 7.4 | 406.3598 [M+NH_4_]^+^ (4), 363.3048 [C_27_H_39_]^+^ (100), 279.2114 [C_21_H_27_]^+^ (6) | C_29_H_40_? |
| 13 | 410.3274 | 638 | 2.1 | C_24_H_44_NO_4_ | -2.2 | 14.7 | 410.3274 [M+NH_4_]^+^ (1), 273.2583 [C_20_H_33_]^+^ (3), 217.1952 [C_16_H_25_]^+^ (5), 203.1796 [C_15_H_23_]^+^ (4), 177.1640 [C_13_H_21_]^+^ (4), 163.1483 [C_12_H_19_]^+^ (8), 149.1326 [C_11_H_17_]^+^ (20), 135.1170 [C_10_H_15_]^+^ (12), 121.1014 [C_9_H_13_]^+^ (32), 109.1015 [C_8_H_13_]^+^ (35), 95.0858 [C_7_H_11_]^+^ (66), 81.0701 [C_6_H_9_]^+^ (100), 69.0702 [C_5_H_9_]^+^ (31) | GGG derivative |
| 14 | 482.3489 | 587 | 2.0 | C_27_H_48_NO_6_ | -1.7 | 2.4 | 482.3489 [M+NH_4_]^+^ (1), 271.2422 [C_20_H_31_]^+^ (100), 215.1796 [C_16_H_23_]^+^ (78), 201.1639 [C_15_H_21_]^+^ (58), 189.1640 [C_14_H_21_]^+^ (48), 175.1480 [C_13_H_19_]^+^ (61), 161.1323 [C_12_H_17_]^+^ (75), 135.1168 [C_10_H_15_]^+^ (71), 121.1014 [C_9_H_13_]^+^ (46), 109.1010 [C_8_H_13_]^+^ (86), 95.0856 [C_7_H_11_]^+^ (79), 81.0698 [C_6_H_9_]^+^ (87), 69.0689 [C_5_H_9_]^+^ (87) | GGG derivative |
| 15 | 307.1520 | 378 | 2.0 | C_17_H_23_O_5_ | 7.2 | 18.4 | 307.1517 [M+H]^+^ (100), 215.1039 [C_14_H_15_O_2_]^+^ (3), 121.1021 [C_9_H_13_]^+^ (6), 115.0363 [C_5_H_7_O_3_]^+^ (8) | C_17_H_22_O_5_ |

^a^ Constructor statistical match factor (comparison of theoretical and experimental isotopic patterns); ^b^ Abbreviations: PC: phosphatidylcholine, DGTA: diacylglycerylhydroxymethyl-*N*,*N*,*N*-trimethyl-*β*-alanine, GGG: geranylgeranylglycerol; ^c^ not observed; ^d^ This identification was confirmed with a purified standard.

# Supplementary Figures

## Figure S1. Global workflow


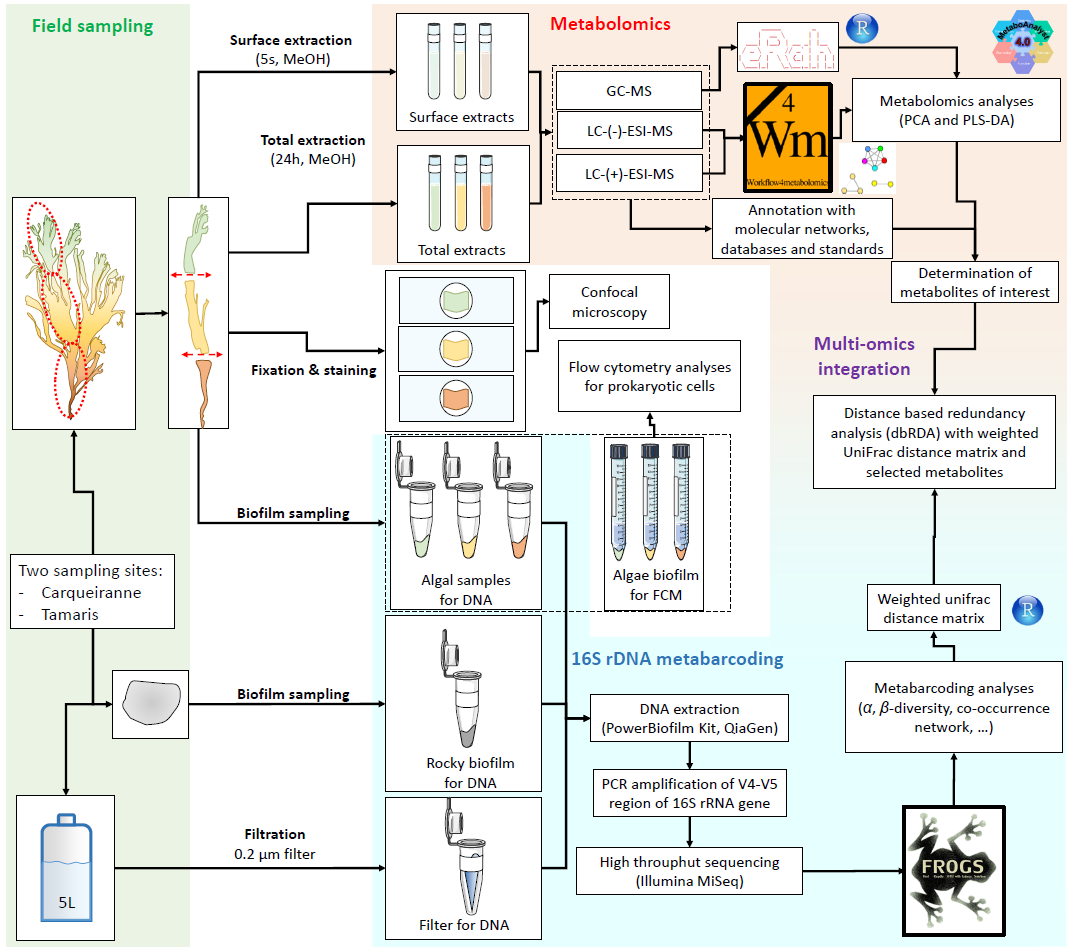


## Figure S2A. Methodologies used for the preparation of fronds and for extraction for metabolomics analysis


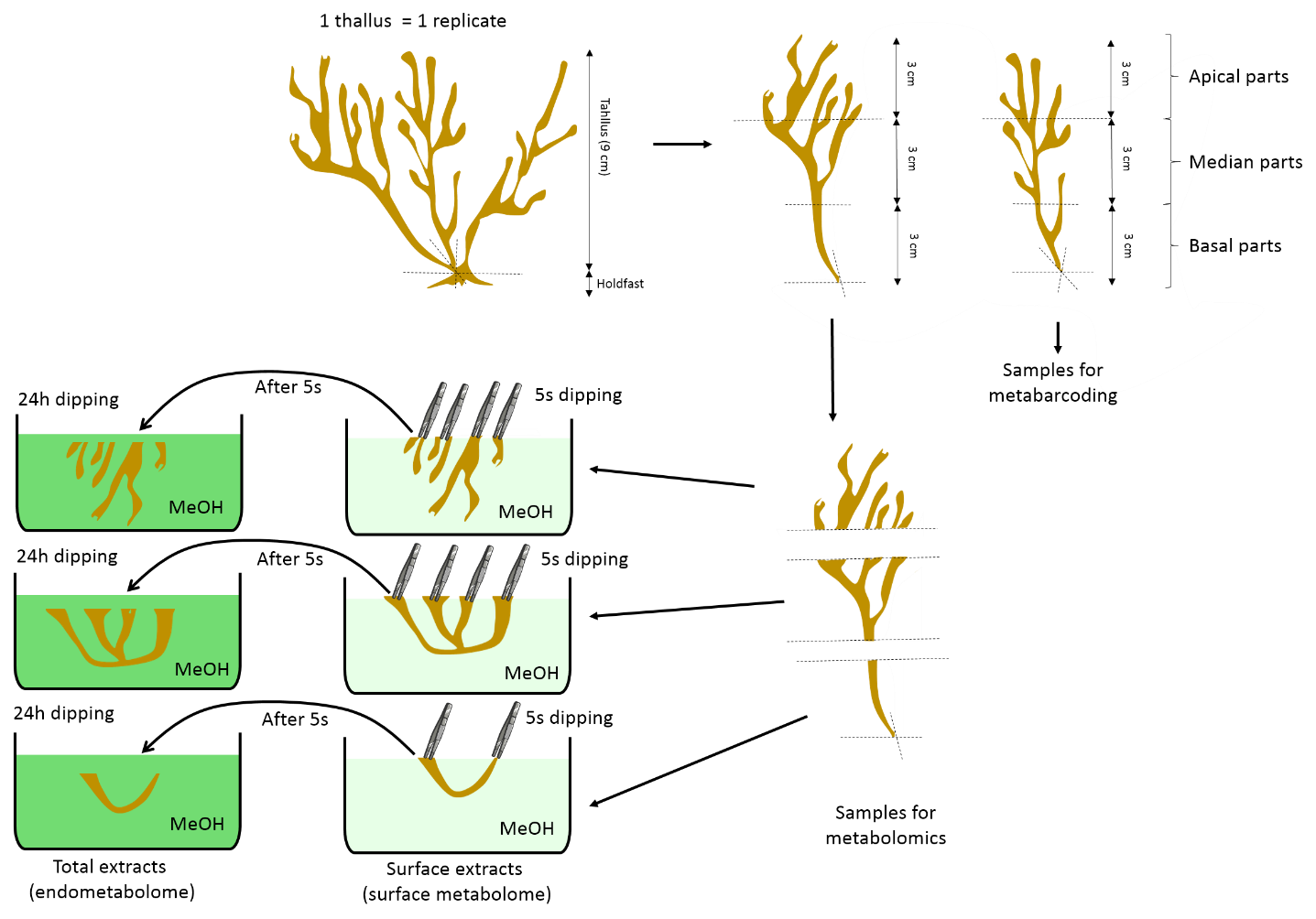


Figure S2B. Description of the separation in three parts (basal, median and apical) of the thalli of *T. atomaria*. Dashed lines represent cut sections separating each part.


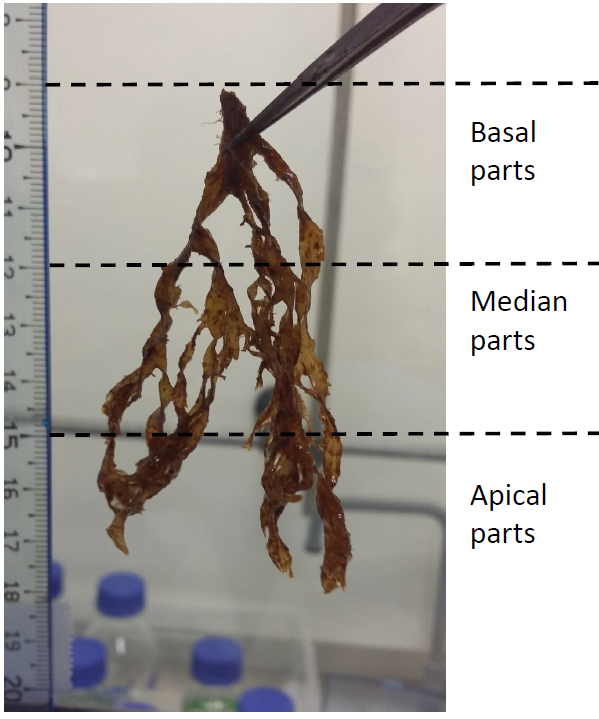


## **Figure S3. Discrimination of heterotrophic prokaryotes by flow cytometry.**

A successive 3-steps workflow was used. A: Samples were first screened for the presence of potential doublets or aggregates. Sample dilution was eventually adjusted in order to keep doublets below 5% of the total signal. B: Particles showing a red fluorescence (FL3) were excluded in order to keep only strict heterotrophs (*i.e.* presenting only the SYBR green-induced fluorescence). C: High side scatter signal harboring particles were excluded in order to enumerate only prokaryotes.


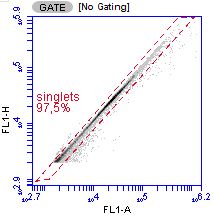

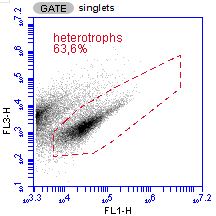

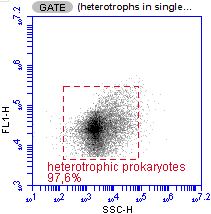


A

B

C

## Figure S4. Confocal microscopy images of basal and apical parts of *T. atomaria* collected at Tamaris.

Images were acquired with a 20X/0.75NA objective. A, B and C corresponded to a first basal part; D, E and F corresponded to images acquired from replicates of basal parts, while G, H and I corresponded to images acquired from an apical part. A, D and G corresponded to images acquired with chlorophyll signal, B, E and H with Dapi signal, and C, F and I with both signals merged. Yellow arrows indicated diatom-like structures observed, blue arrows indicated filamentous bacteria-like structures.


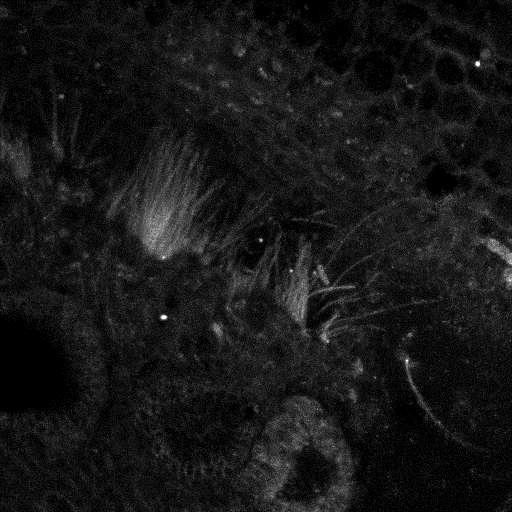

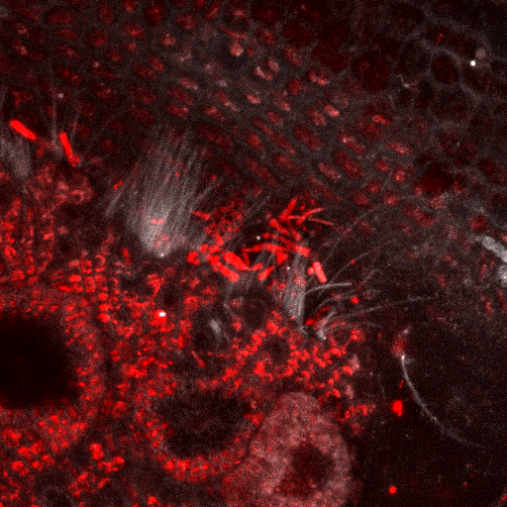

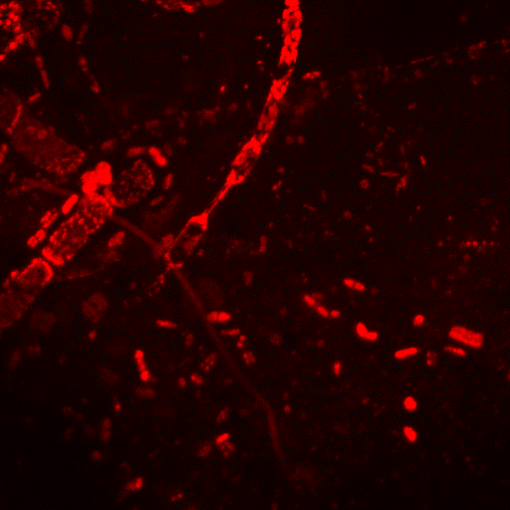

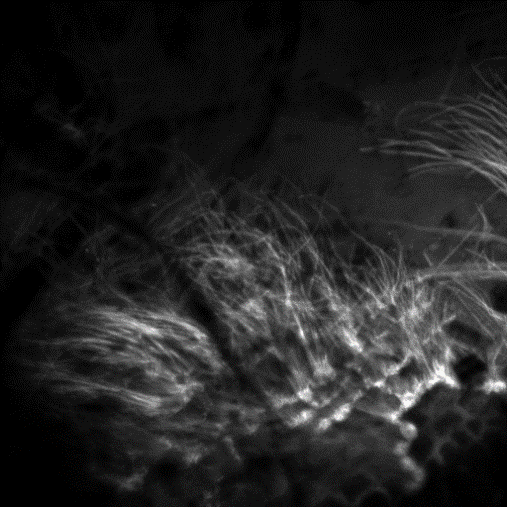

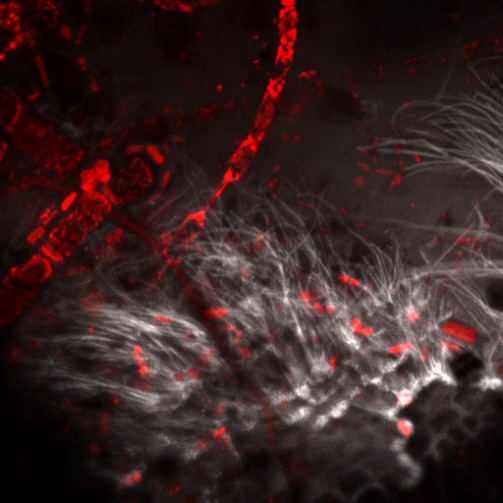

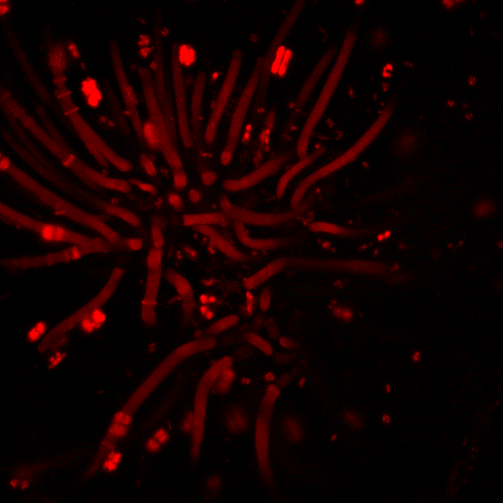

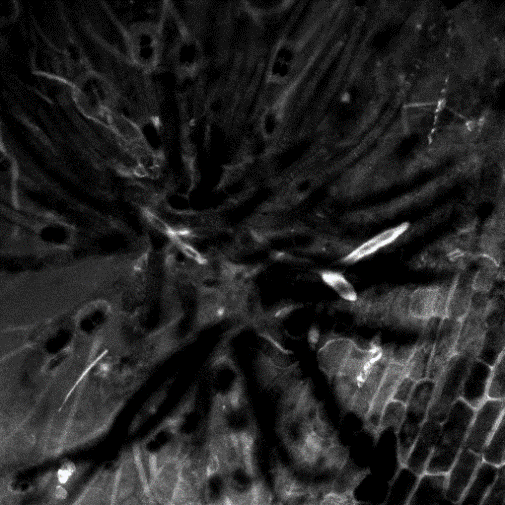

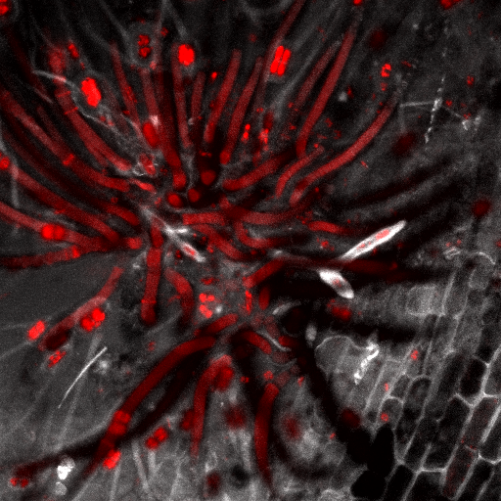

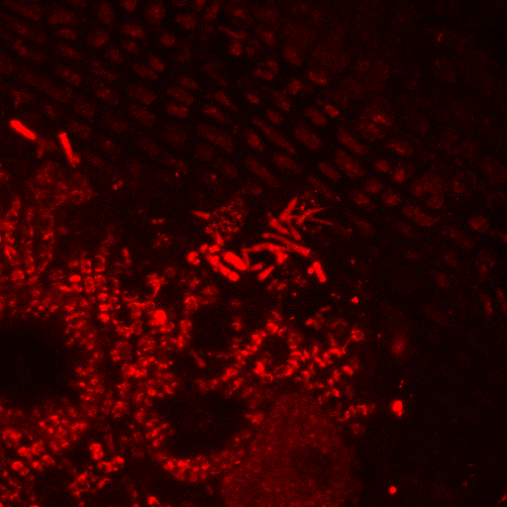


50 µm

50 µm

50 µm

Chlorophyll

Dapi

Chlorophyll + Dapi

A

C

B

D

F

E

G

I

H

## Figure S5. Rarefaction curves obtained after data processing of 16S rRNA gene sequences.

Red, yellow, green, grey and blue lines represented respectively samples from basal (.B) , median (.M) and apical (.A) algal parts, rocky biofilms and water samples. Dashed lines represented Carqueiranne samples (C.) and full lines represented Tamaris samples (T.).


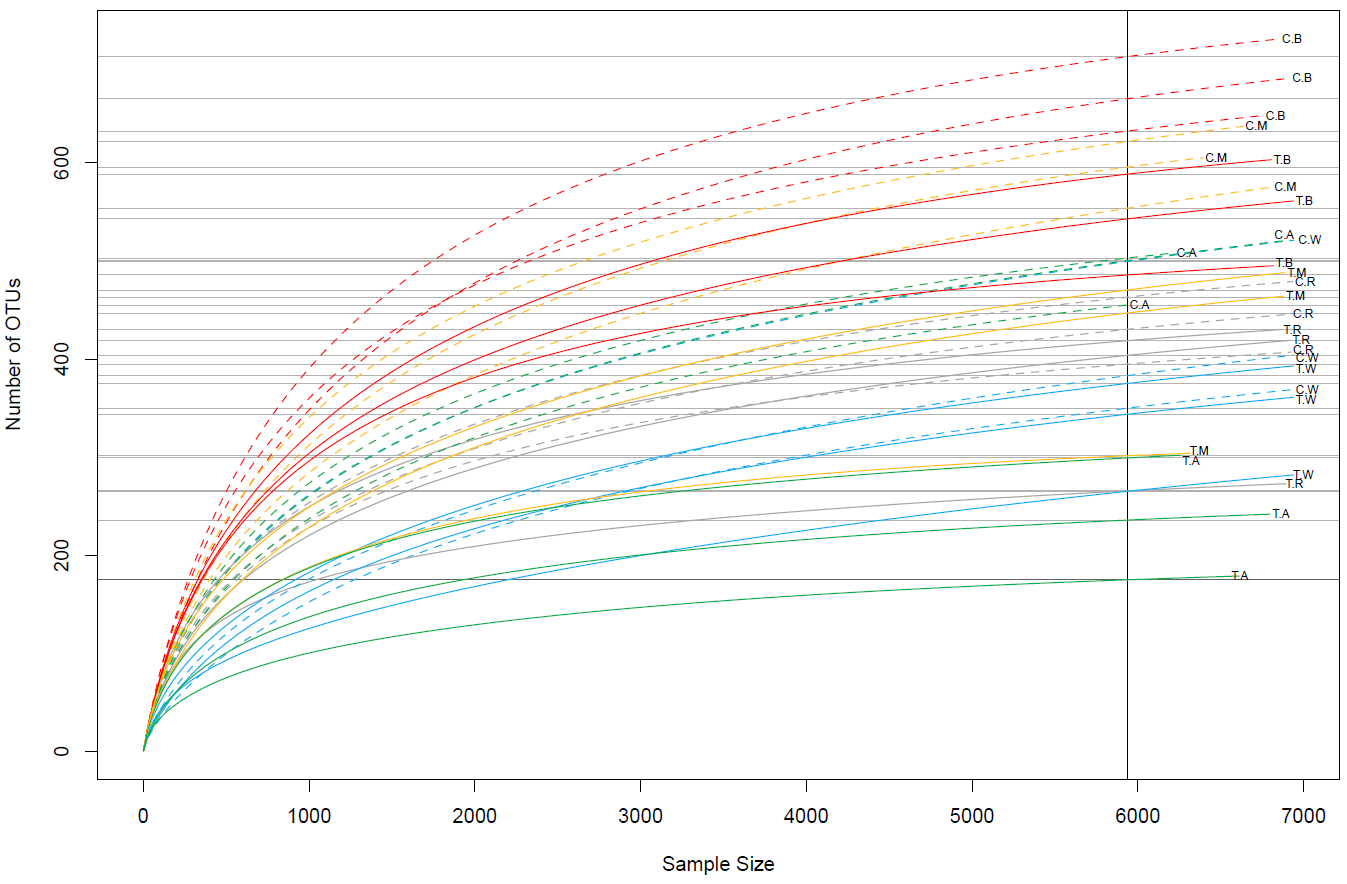


## Figure S6. Unifrac distances between rocky biofilms (R) and the three thallus parts [basal (B), median (M) and apical parts (A)], or between seawater samples (W) and the three thallus parts.


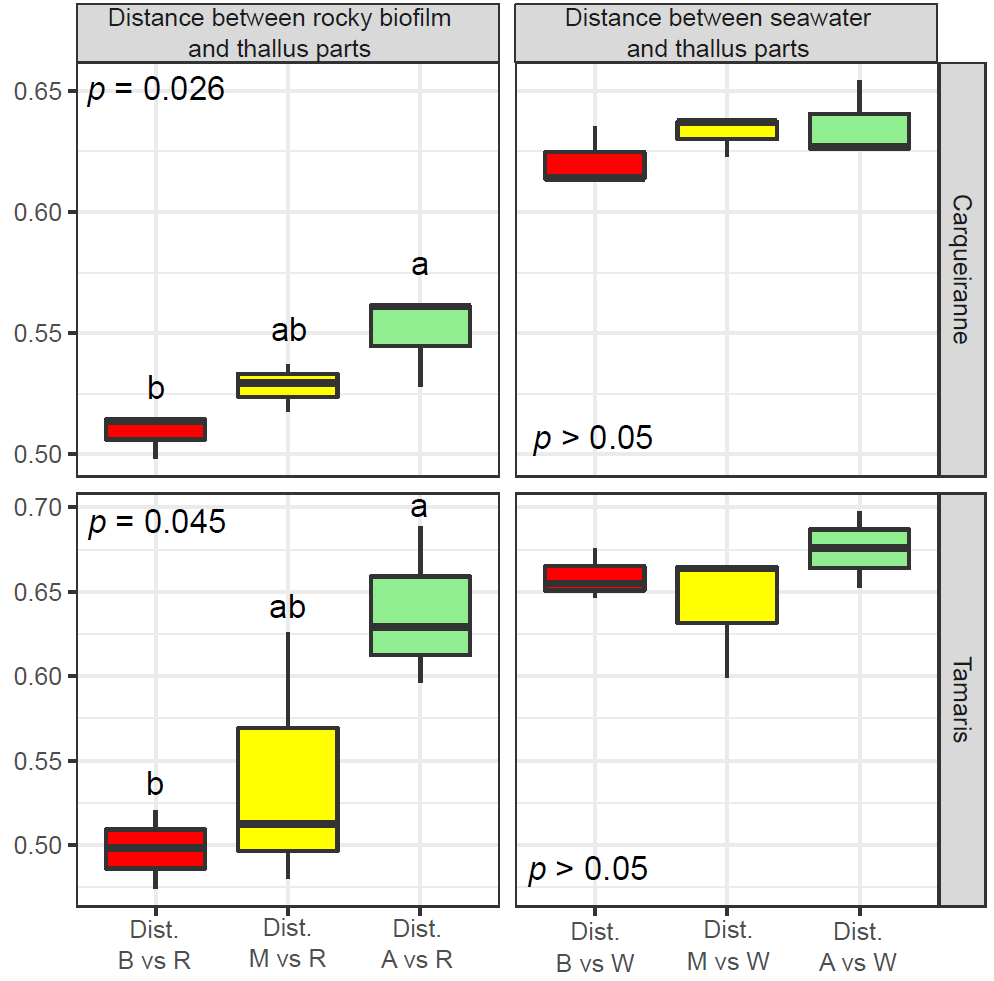


## Figure S7. Cladogram obtained from the LEfSe analysis built with the 16S rRNA gene dataset of bacterial communities at the surface of *T. atomaria*, in rocky biofilms and in seawater at Tamaris.


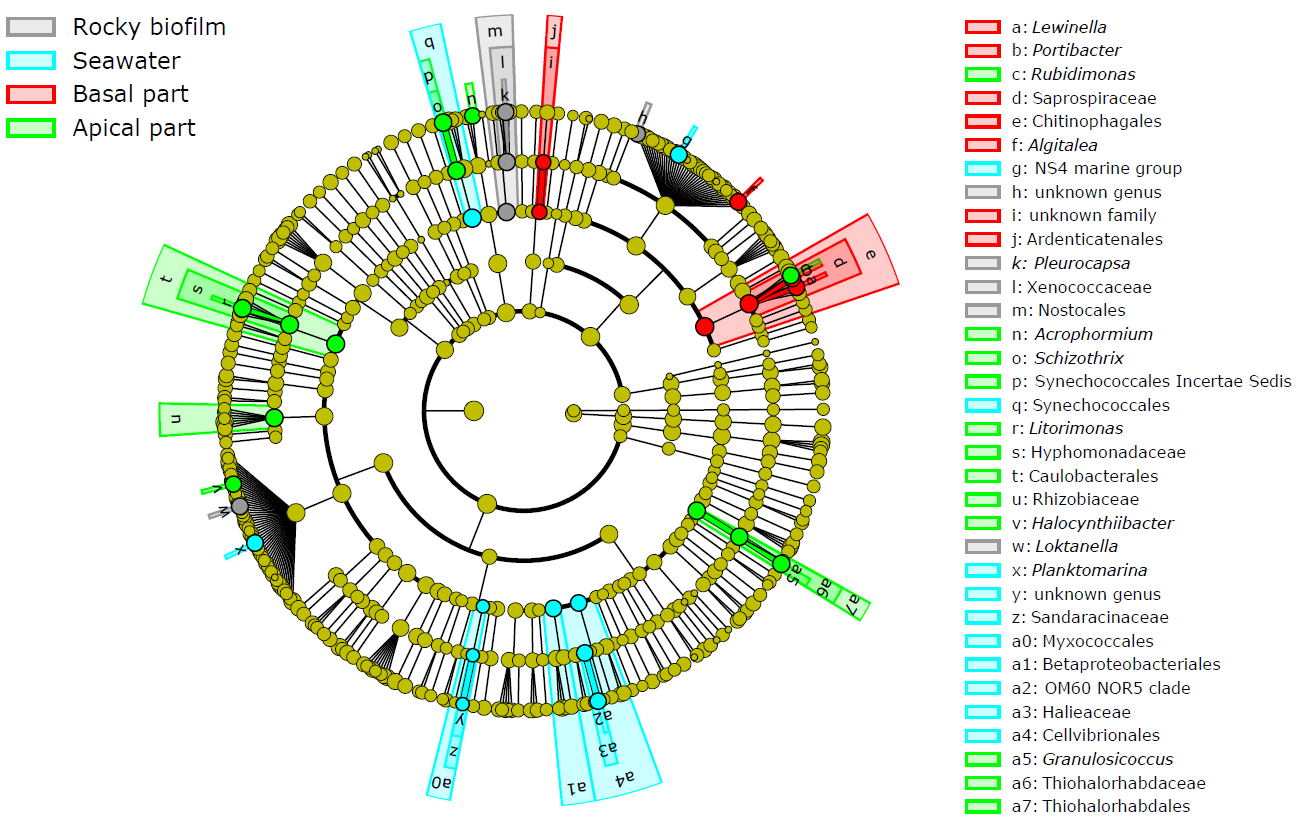


## Figure S8. Cladogram obtained from the LEfSe analysis built with the 16S rRNA gene dataset of bacterial communities at the surface of *T. atomaria*, in rocky biofilms and in seawater at Carqueiranne.


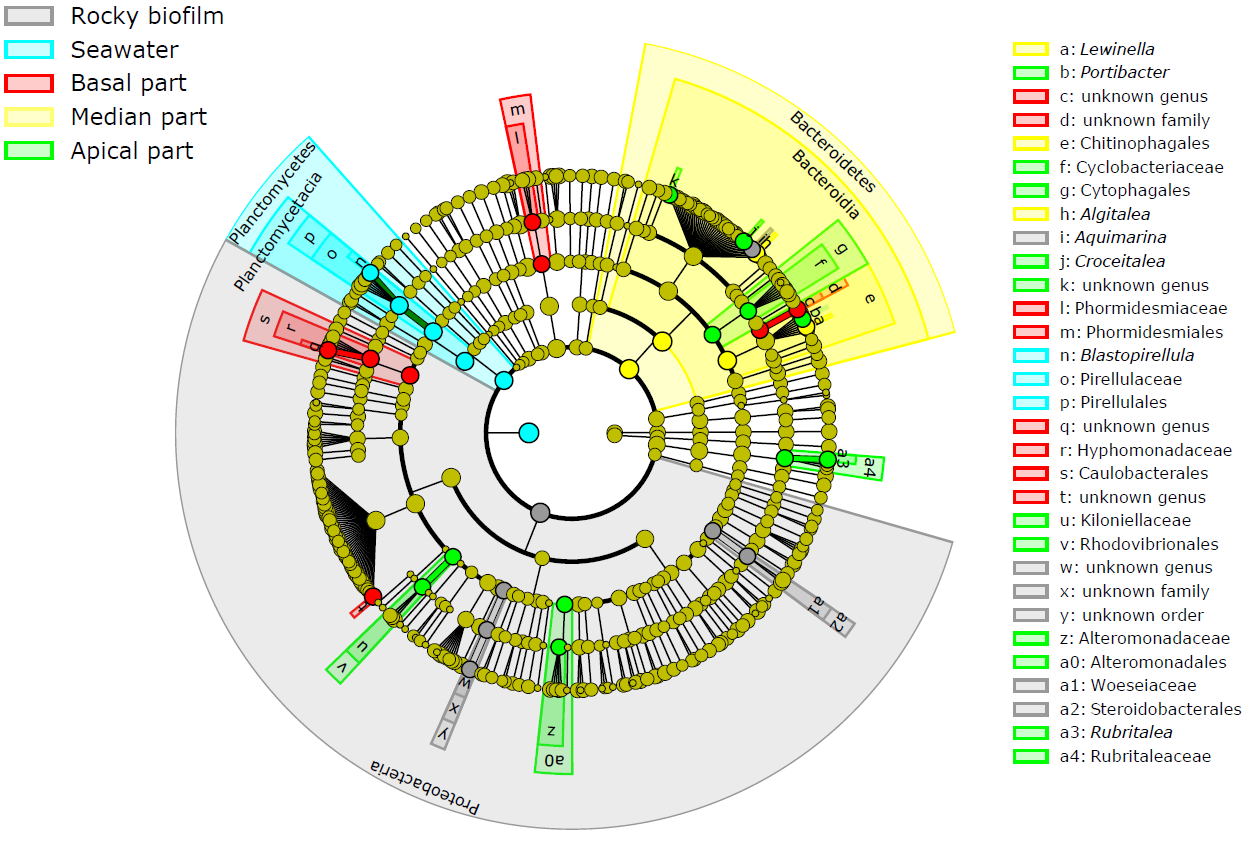


## Figure S9. Co-occurrence network of OTUs from the 16S rRNA gene dataset of bacterial communities at the surface of *T. atomaria*, in rocky biofilms and in seawater at Tamaris

Pie-chart inside each node revealed the distribution of each OTU across the different sample groups (Red: basal part, yellow: median part, green: apical part, grey: rocky biofilm, blue: seawater). Thickness of edges between each node was proportional to correlation or dissimilarity distances. The size of each node was proportional to the relative percentage of sequences of each OTU in all samples.


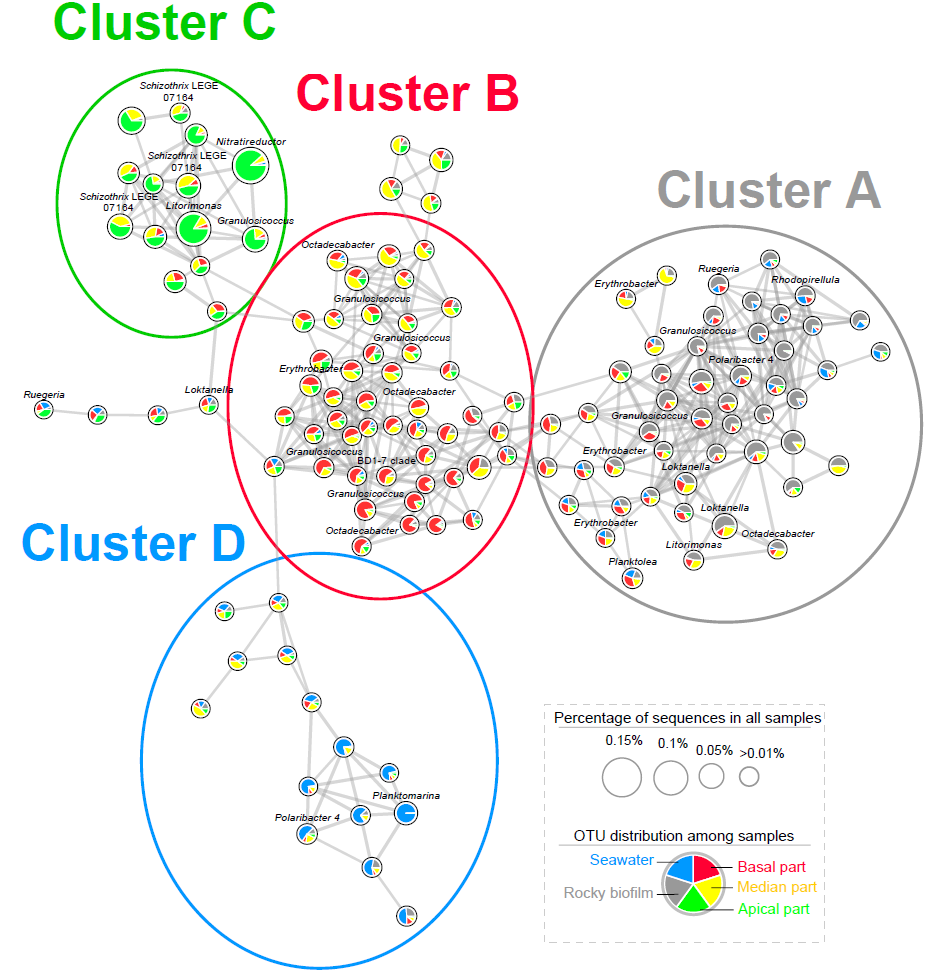


##

## Figure S10. Venn diagrams showing shared percentages of sequences between: (i) the different thallus parts and rocky biofilms and (ii) the different thallus parts and sea water.


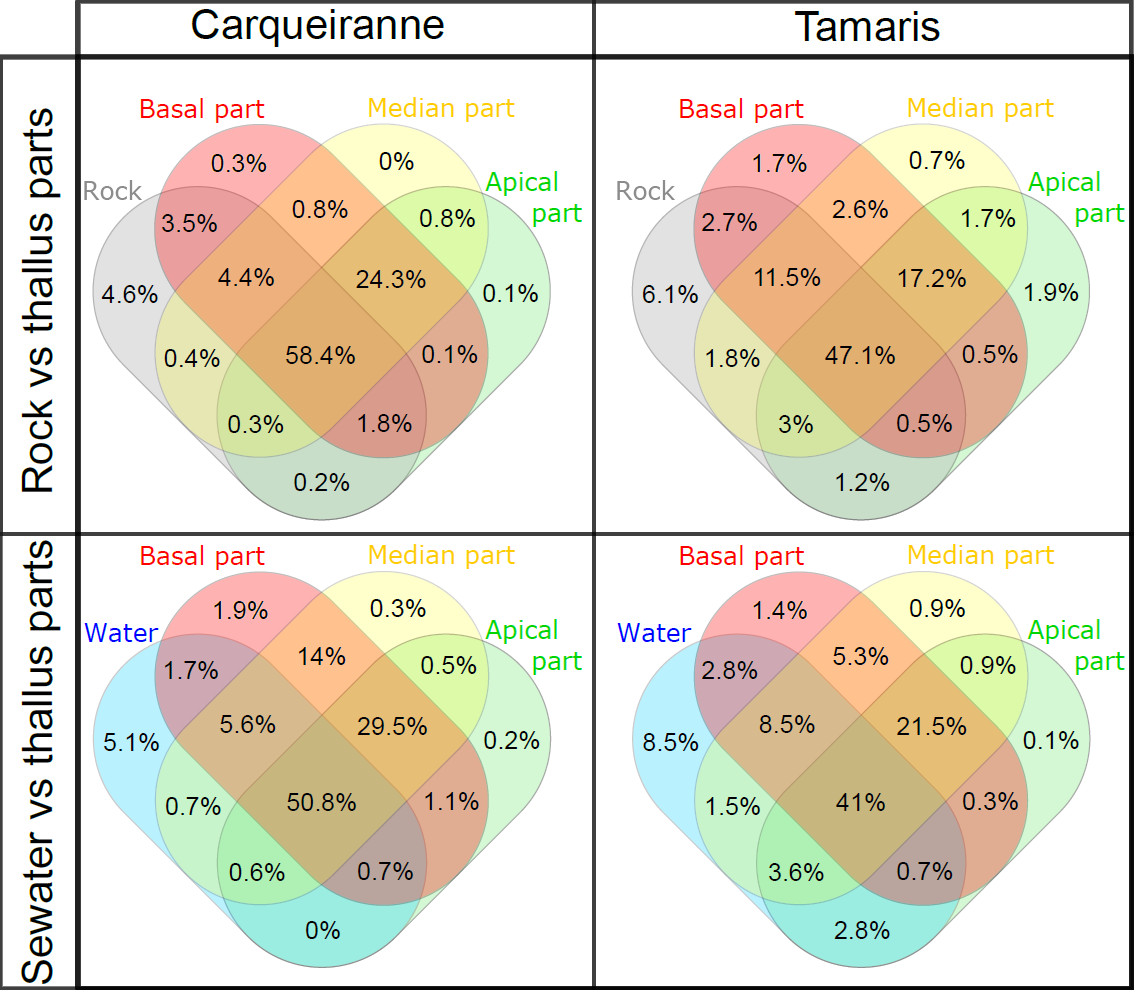


## Figure S11. Chemodiversity indexes (Shannon) of surface extracts of *T. atomaria* (two sites: Carqueiranne and Tamaris; three algal parts: basal, median and apical) analyzed by LC-(+)-ESI-MS, LC-(-)-ESI-MS and GC-MS

Boxplots in red, yellow and green, respectively labeled (“B”, “M” and “A”) represented basal, median and apical algal parts.


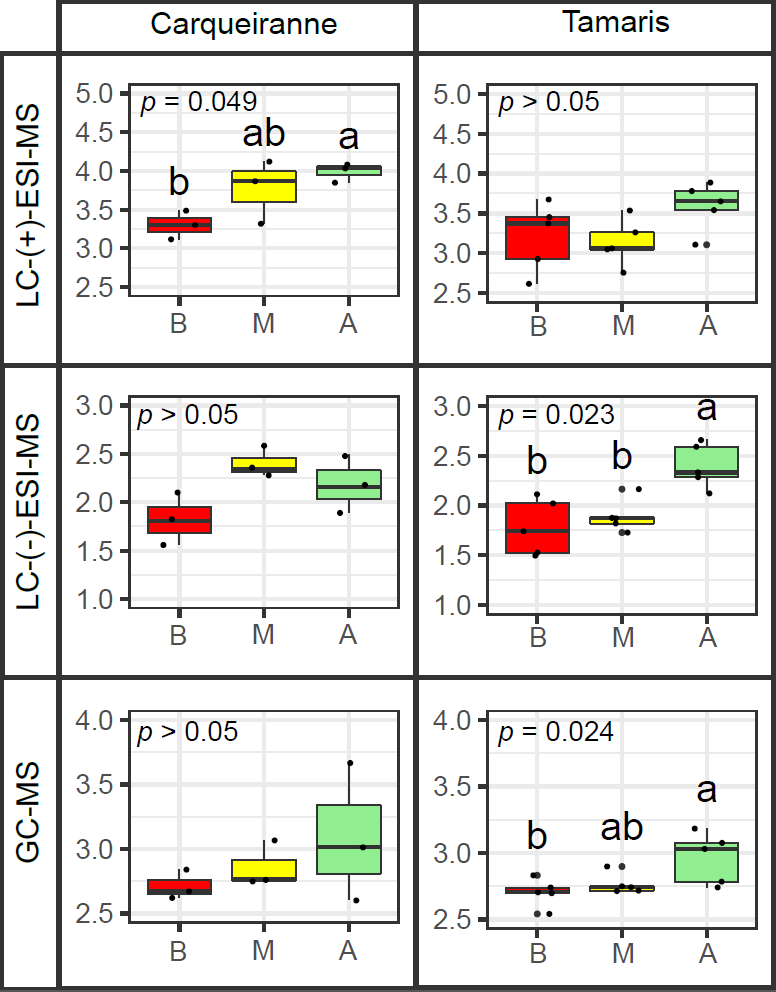


## Figure S12. PLS-DA plots of GC-MS, LC-(-)-ESI-MS and LC-(+)-ESI-MS metabolomics analyses of surface and total extracts of *T. atomaria* (two sites: Carqueiranne and Tamaris; three algal parts: basal, median and apical).


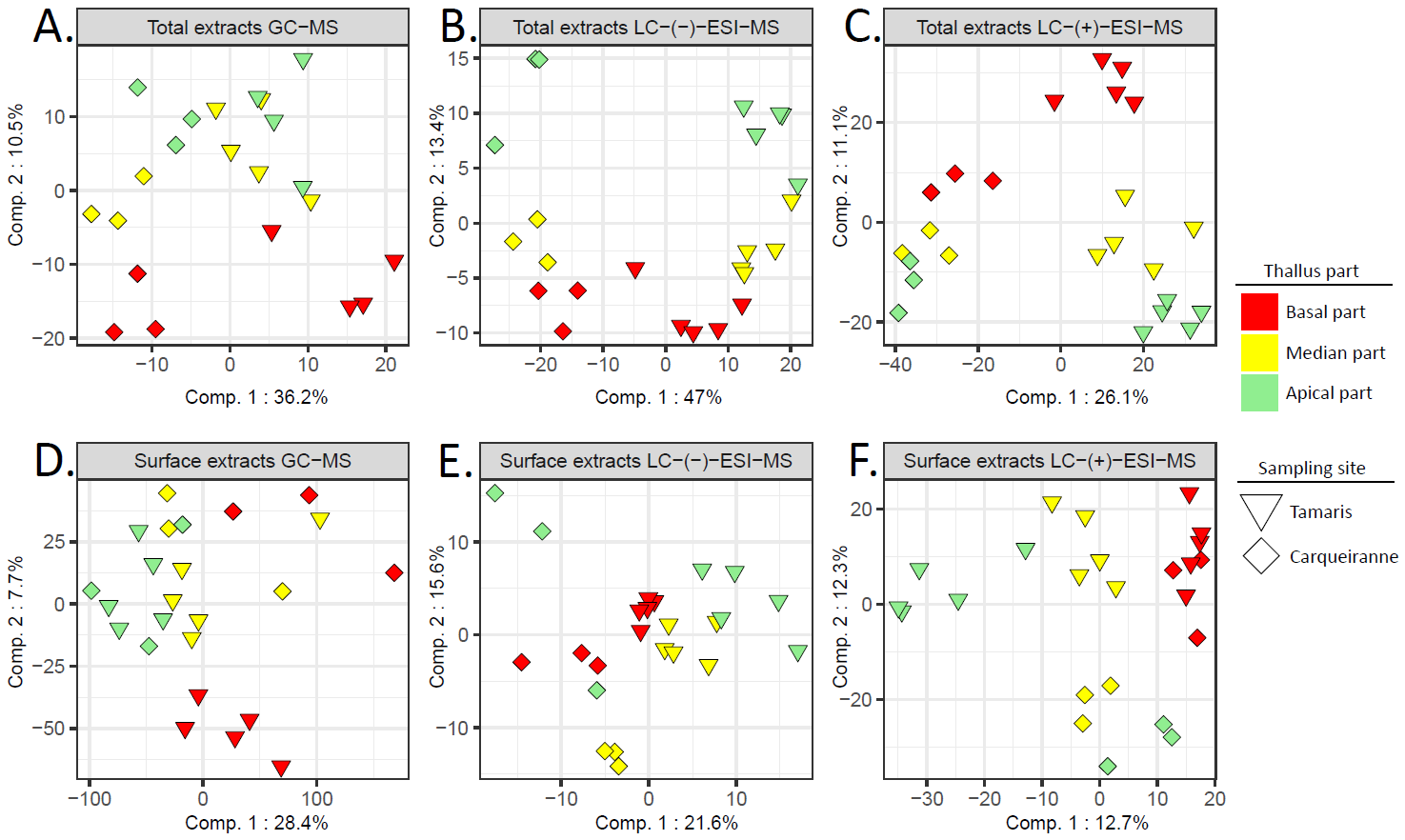


## Figure S13. Molecular network for LC-(+)-ESI-MS dataset annotation.

Node colors were chosen according to their annotation. Node shapes were chosen according to their level of annotation (Schymanski et al., 2014; diamonds: level 1, squares: level 2, triangles: level 4 with a proposed molecular formula, circles: level 4 without molecular formula). Thickness and transparency of edge between two nodes was proportional to the cosine score (CS). Only clusters with at least three nodes were represented.


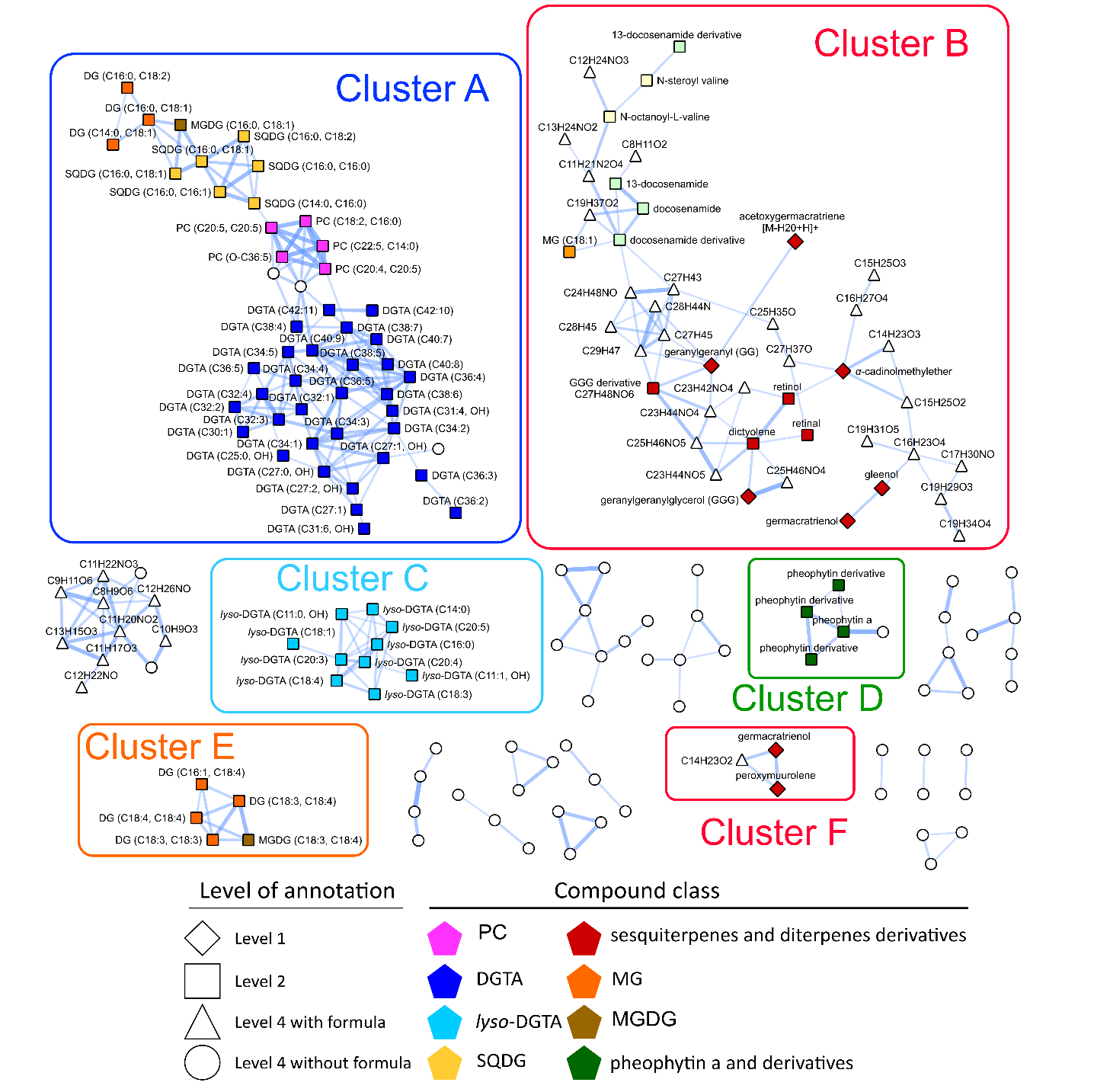


## Figure S14. Molecular network for LC-(-)-ESI-MS dataset annotation.

Node colors were chosen according to their annotation. Node shapes were chosen according to their level of annotation (Schymanski et al., 2014; squares: level 2, circles: level 4). Thickness and transparency of edge between two nodes was proportional to the cosine score (CS). Only clusters with at least three nodes were represented.


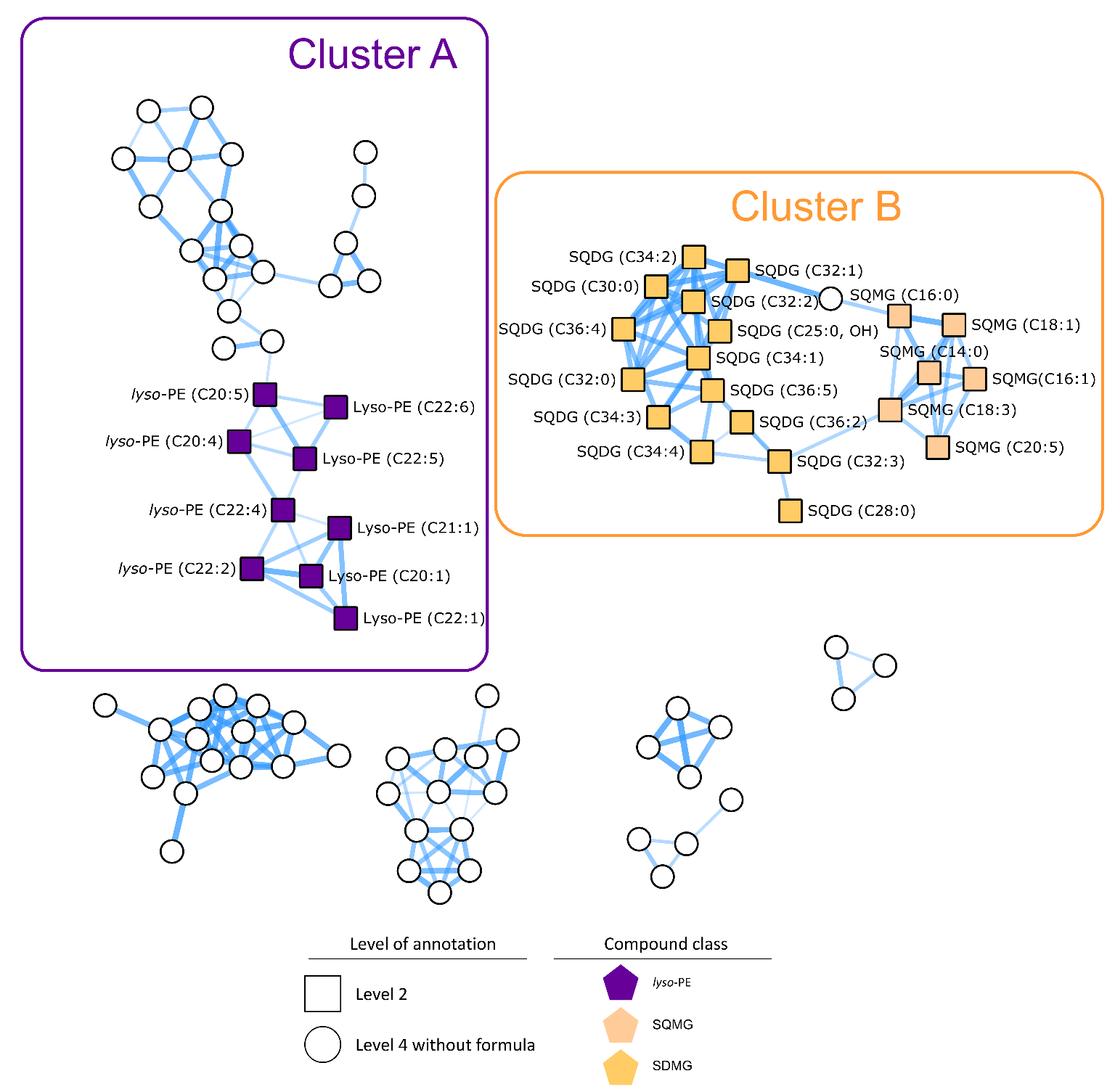


Figure S15. Heatmap of sesquiterpenes variations across samples identified in the GC-MS dataset.

*Confirmed through purified standards.

## Figure S16. Heatmap of annotated compounds variations identified in the LC-(+)-ESI-MS dataset (1/2).


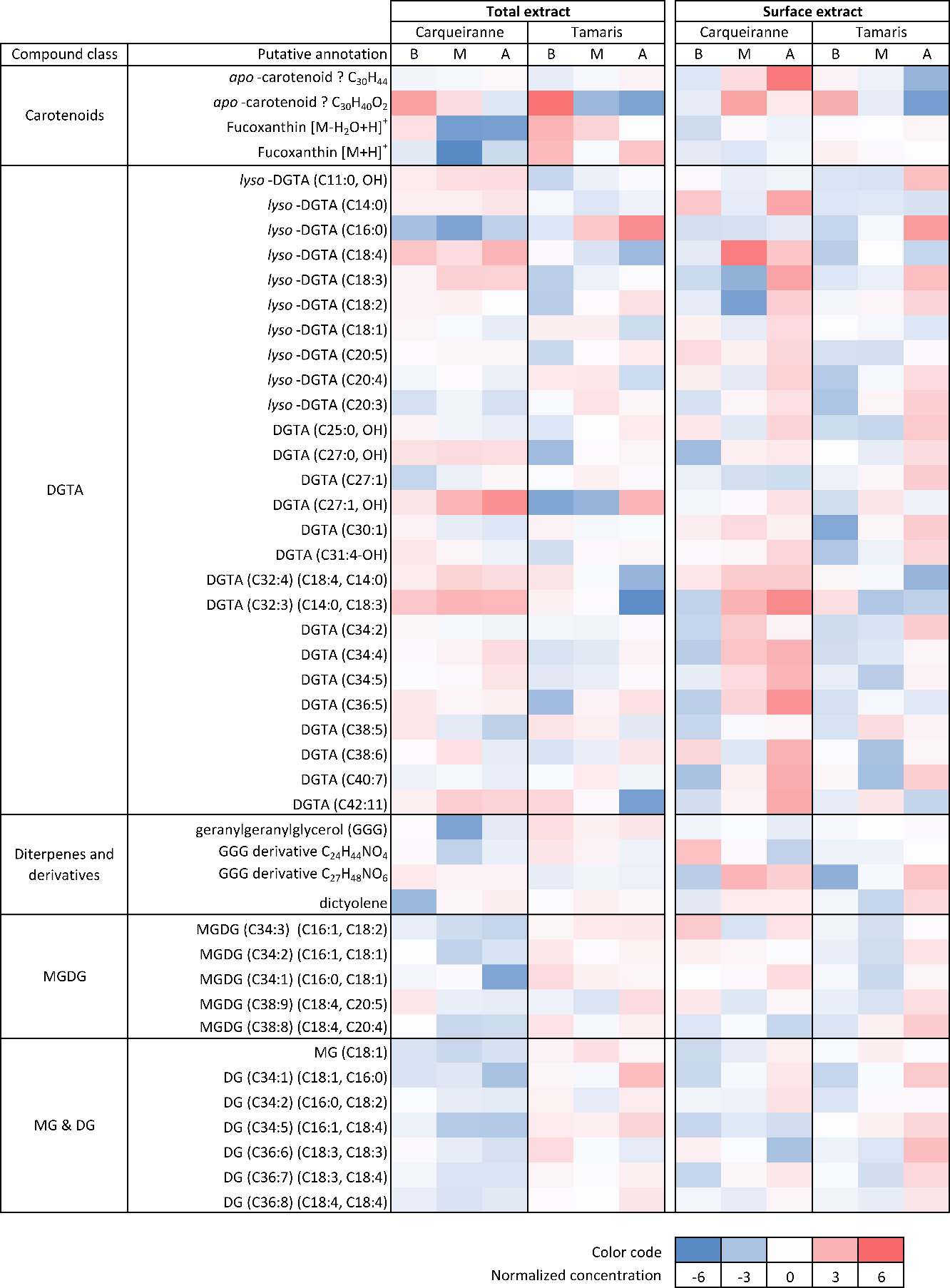


## Figure S16. Heatmap of annotated compounds variations identified in the LC-(+)-ESI-MS dataset (2/2).


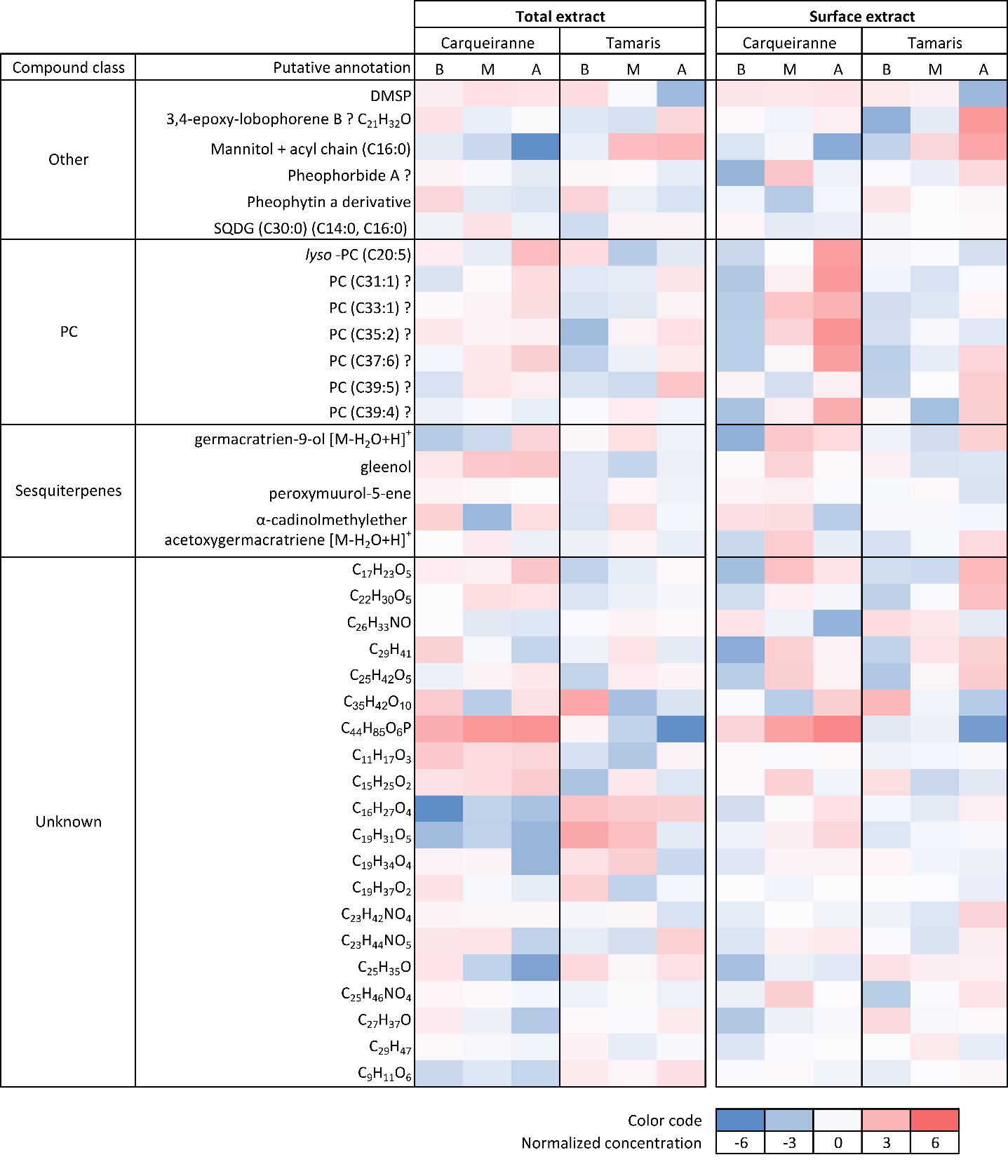


## Figure S17. Heatmap of annotated compounds variations identified in the LC-(-)-ESI-MS dataset.
